# A time-dependent parameter estimation framework for crop modeling

**DOI:** 10.1038/s41598-021-90835-x

**Published:** 2021-06-01

**Authors:** Faezeh Akhavizadegan, Javad Ansarifar, Lizhi Wang, Isaiah Huber, Sotirios V. Archontoulis

**Affiliations:** 1 Department of Industrial and Manufacturing Systems Engineering, Iowa State University, Ames, IA 50011 USA; 2 Department of Agronomy, Iowa State University, Ames, IA 50011 USA

**Keywords:** Plant sciences, Machine learning

## Abstract

The performance of crop models in simulating various aspects of the cropping system is sensitive to parameter calibration. Parameter estimation is challenging, especially for time-dependent parameters such as cultivar parameters with 2–3 years of lifespan. Manual calibration of the parameters is time-consuming, requires expertise, and is prone to error. This research develops a new automated framework to estimate time-dependent parameters for crop models using a parallel Bayesian optimization algorithm. This approach integrates the power of optimization and machine learning with prior agronomic knowledge. To test the proposed time-dependent parameter estimation method, we simulated historical yield increase (from 1985 to 2018) in 25 environments in the US Corn Belt with APSIM. Then we compared yield simulation results and nine parameter estimates from our proposed parallel Bayesian framework, with Bayesian optimization and manual calibration. Results indicated that parameters calibrated using the proposed framework achieved an 11.6% reduction in the prediction error over Bayesian optimization and a 52.1% reduction over manual calibration. We also trained nine machine learning models for yield prediction and found that none of them was able to outperform the proposed method in terms of root mean square error and R^2^. The most significant contribution of the new automated framework for time-dependent parameter estimation is its capability to find close-to-optimal parameters for the crop model. The proposed approach also produced explainable insight into cultivar traits’ trends over 34 years (1985–2018).

## Introduction

Crop models build upon the physiological understanding of plant response to the soil, weather, and management conditions. This understanding is approximated with a series of non-linear equations while the effects of different soil properties and cultivars are captured by different parameter values (input parameters to the model)^1,2^. Presently, there are many crop models including APSIM^3^, DSSAT^4^, EPIC^5^, FASSET^6^, SWAP and WOFOST^7^, SOYGRO^8^, and crop modeling groups^9^. Different models share similar modeling principles but differ in equations and parameter values used. Crop models are widely used in testing crop management strategies^10^, forecasting crop yield^11^, evaluating cultivars in breeding programs^12^, and exploring climate change impacts on productivity^13^. For example, Archontoulis et al.^14^ and Togliatti et al.^11^ used the APSIM crop model to predict crop yields in the US Corn Belt. Dumont et al.^15^ tested the crop yield performance of the crop model under two different weather scenarios (stochastically generated climatic data and the mean climate data).

Mechanistic crop models aim to mimic natural processes by simulating crop performance for each set of input values. When the model is used outside of a tested environment, a parameter calibration (i.e., change of cultivar or soil input values) is typically needed to improve the simulation output and be closer to observations^16^. The more parameters that need to be calibrated, the more complex the calibration process is. Manual calibration by trial and error^17^ is commonly used to estimate parameter values. Crop modelers have developed protocols (step by step approaches) in which calibration starts with phenological parameters, then continues with biomass production and partitioning related parameters^14,18,19^. Calibration processes need to run a crop model multiple times using an effective and iterative procedure to evaluate each parameter’s effect on crop model outputs^20^.

Parameter calibration algorithms used to improve crop models’ performance have been classified into two categories: local and global methods^21^. Local methods, called simple derivative-based methods, evaluate crop model outputs’ response to variation one parameter at a time while keeping the other parameters fixed^22^. The implementation of these methods is relatively easy with a low computational cost. However, they suffer several drawbacks, including the inability to specify impacts of interactions between parameters on outputs and high dependency on the parameter’s base value^21^. On the other hand, global methods have been developed to overcome these drawbacks by evaluating the impacts of the entire multi-dimensional parameter space on crop model outputs. These methods provide a more comprehensive view of the sensitivity analysis of parameters on outputs. Several global sensitivity analysis methods have been proposed to calibrate the parameters in crop models, including Fourier amplitude sensitivity test (FAST)^23^, extended FAST^24^, multi-normal approximations^25^, simulated annealing^26^, shuffled complex evolution method^27^, least squares^28^, regression-based model^29^, interaction-based model^30^, non-parametric smoothing^31^, Markov chain Monte Carlo parameter estimation^20,32–35^, generalized likelihood uncertainty estimation^32^, multi-model ensembles^36^, maximal conditional posterior distribution^37^, hybrid metropolis Hastings Gibbs algorithm^38^, differential evolution adaptative metropolis algorithm^39,40^, Bayesian model^33,35,41–44^, Bayesian optimization (BO)^45^, and Bayesian-based multilevel factorial analysis^46^. FAST methods have fast convergence with an acceptable computing cost and less accuracy for non-linear and complex models^47,48^. The shuffled complex evolution, simulated annealing, and differential evolution try to find optimal or close-to-optimal values for parameters and do not provide any information about the parameter estimates’ confidence^39^. Non-parametric smoothing can be applied to quantify the main effects of parameters on the response variable when there are low order interactions between parameters and response variable. Non-parametric smoothing and approximation models were not reliable for non-linear and complex models^10,49^. A Bayesian model needs a definition of a prior distribution where, in most cases, there is no reliable prior knowledge. Monte Carlo-based methods are computationally intensive and fail to provide adequate sample density from solution space when the number of parameters increases^50^.

In capturing historical trends in crop models like maize yield increase from 1985 to 2018 in the US Corn Belt, cultivar parameters need to be time-dependent to reflect changes in crop cultivars due to plant breeding or environment^51–60^. Using a static set of cultivar parameter values will not capture the trend of observed outcome or simulate how nature produces output from input over time in the US Corn Belt and other regions^61,62^. Development of time-dependent parameters is challenging because of the lack of specific data as well as the absence of a robust calibration framework for time-dependent parameters. The more time-dependent parameters that need to be calibrated, the more complex the calibration process is. To our knowledge, most of the previous model calibration exercises using optimization algorithms were not dealing with the time-dependent parameter values^16,17,20,39,49^. Integrating prior agronomic knowledge with an optimization model to develop a constrained model can reduce the prediction error to zero, but that would be over-fitting on the observed data set. Hence, the calibration structure must be designed in such a way to improve crop model performance and prevent the over-fitting problem. Moreover, this structure must follow some prior agronomic knowledge such that parameter trends have to follow a specific pattern over time but spatially may differ from location to location.

The goal of this paper is to develop a new time-dependent parameter calibration method for crop modeling towards improving the simulation accuracy. We designed a new automated and efficient framework and a parallel Bayesian optimization (PBO) algorithm to estimate time-dependent parameters from limited data that could integrate the prior agronomic knowledge constraints. This research describes the new algorithm and its prediction accuracy and further compares prediction accuracy against the prediction using a Bayesian algorithm and a manual calibration approach. We considered historical yield data (1984 to 2019; 34 years) from 25 locations across the US Corn Belt. Finally, we interpret time-dependent parameter values towards explaining historical yield increase in this region.

## Problem definition

**Figure 1:**
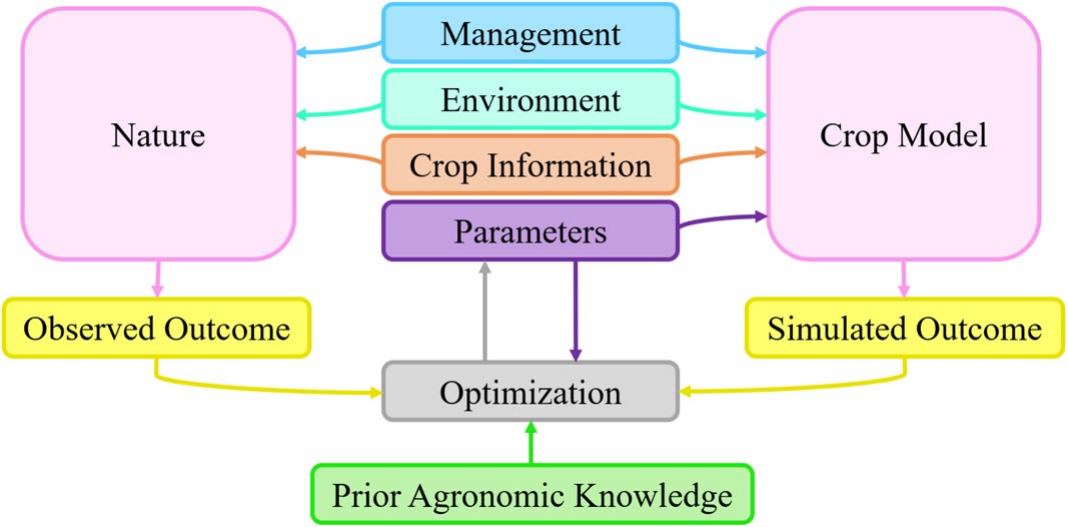
The structure of parameter calibration in the simulation of crop growth and development.

Crop models simulate how nature produces outcome performance from appropriate inputs using the physiological understanding of plant processes^1,2^; however, not all input parameters are available to inform crop models to mimic nature accurately. In capturing historical trends (e.g., yield increases) by crop models, which is the product of concurrent changes in cultivar, management, technological gains, equipment, and environment, the main challenge is to derive time-dependent parameters’ values resulting from changes in plant traits with years of plant breeding or environment^58–60^. Weather and management data are typically available. In terms of cultivar, there are good records on grain yields but not on the underlying traits, which are necessary inputs to crop models. The challenge is to approximate the crop model’s parameters with limited data available. That is why parameter calibration is an essential job in using crop models. The structure of parameter calibration in crop growth and development simulation is illustrated in Fig. 1. Although the manual and automated parameter calibration simulate outcomes that may be very consistent with observed outcomes, they could not integrate prior agronomic knowledge or time dependency of parameters within their structures. Unconstrained calibration approaches at least produce the marginal solution for the future because they ignore the time-dependency between parameters and prior agronomic knowledge during their processes. From an agronomic point of view, a constrained calibration can satisfy the reasonable trend for parameters.

## Method

**Figure 2:**
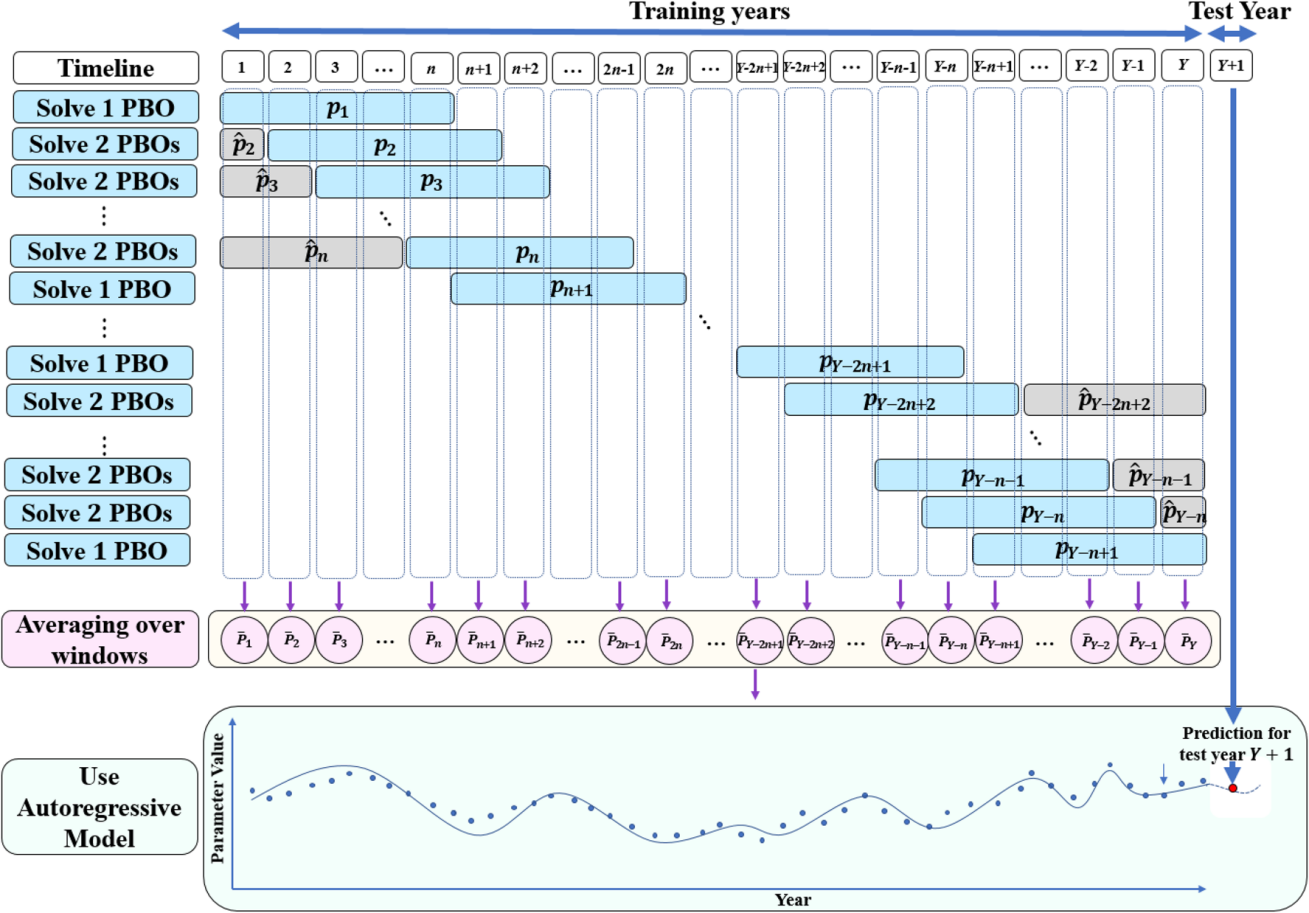
The proposed framework of time-dependent parameter calibration.

The proposed framework for time-dependent parameter calibration is shown in Fig. 2. The training data set from year 1 to year *Y* was divided into two different time-windows. Time-window *p* with $n>1$ years denotes main time-window and time-window ${\hat{p}}$ with less than *n* years denotes auxiliary time-window. The main time-windows are defined so that two successive time-windows have $n-1$ years overlap. For example, the first main time-window contains years 1 to *n*, where the second one is from year 2 to year $n+1$ and so on. The final main time-window covers year $Y-n+1$ to year *Y*. Some main time-windows have auxiliary time-windows. To estimate the parameters based on *n* time-windows for each year, the auxiliary time-window is defined for the first and the last training years. After defining the time-windows, we used the PBO as the optimization model in the structure of parameter calibration shown in Fig. 1 to optimize each window’s parameters to minimize the crop model’s prediction error. Then, to calculate the best combination of parameters at year *y* (${\bar{P}}_y$), we take a weighted average of parameters over windows that year *y* is covered by them as follows:

$$\begin{aligned} {\bar{P}}_{y}= {\left\{ \begin{array}{ll} \sum _{i=1}^{y} \frac{np_{i}}{\sum _{i=1}^{y}n+\sum _{i=y+1}^{n}(i-1)}+\sum _{i=y+1}^{n} \frac{(i-1){\hat{p}}_{i}}{\sum _{i=1}^{y}n+\sum _{i=y+1}^{n}(i-1)}, &{} \text {if}\ y < n\\ \sum _{i=y-n+1}^{y} \frac{p_{i}}{n}, &{} \text {if}\ n \le y \le Y-n+1\\ \sum _{i=y-n+1}^{Y-n+1} \frac{np_{i}}{\sum _{i=y-n+1}^{Y-n+1}n+\sum _{i=Y-2n+2}^{y-n}(Y-n-i+1)}+\sum _{i=Y-2n+2}^{y-n}\frac{(Y-n-i+1){\hat{p}}_{i}}{\sum _{i=y-n+1}^{Y-n+1}n+\sum _{i=Y-2n+2}^{y-n}(Y-n-i+1)}, &{} \text {if}\ y> Y-n+1 \end{array}\right. } \end{aligned}$$

Taking an average of *n* time-windows for each year tries to reduce variance in parameter estimation because it avoids over-fitting. To obtain a consistent trend for each parameter, we fit the autoregressive model^63^ on the average value of the parameters during the simulation period because at most the two successive years have $n-1$ common time-windows. Also, applying the Augmented Dickey-Fuller test^64^ reveals that the trend of the parameter over simulation periods has autocorrelation. The parameters’ trend cannot reject the null hypothesis that a unit root is present in a time series sample. The notation *AR*(*t*) indicates an autoregressive model of order *t*. Order *t* means future value depends on how many years before year *y* need to be considered.

$$\begin{aligned} {\bar{P}}_{y}=\beta _0+\sum _{i=1}^{t} \beta _i {\bar{P}}_{y-i}+\varepsilon _{y} \end{aligned}$$

where $\beta _0$, $\beta _i$, and $\varepsilon _{y}$ are constant, the model’s parameters, and white noise at year *y*, respectively. For each parameter, we can use the partial autocorrelation function^65^ to determine the optimal order in the autoregressive model. Then we use this autoregressive model to predict parameter values for test year $Y+1$ and run APSIM with them to simulate crop yield performance for test year $Y+1$. In this structure, the autoregressive model is used, while this structure can be extended to other types of time series models. Moreover, the number of years in the main time-window (*n*) shows the trade-off between bias and variance in predicting parameters trend over simulation periods. Increasing *n* is to increase the bias and reduce the variance because of under-fitting on the training set and vice versa. Parameter *n* is the key hyper-parameter that can affect the performance of the algorithm. Optimizing *n* can minimize the summation of bias and variance. We tried different values for *n* in the case study, and $n=5$ resulted in the best performance.

### Parallel Bayesian optimization

This section proposes an extended version of BO^66^ to improve the time-dependent parameter calibration structure in the proposed framework (see Fig. 2). We briefly review the classic BO method and the key statistical and optimization methods on which it relies in the Appendix 1. We use BO because it is one of the most successful approaches in optimizing calibration problems^67^. BO suffers from some limitations, and thus we modify this algorithm. First, BO has a high sensitivity to the type of acquisition function as an objective function and the nature of the gaussian procedure (GP), including kernel types and kernel hyperparameters^68^. Second, BO loses its efficiency in exploration and exploitation as the input dimensions increase. Third, the acquisition function is often difficult to optimize, and its performance depends on an optimizer to search the surface.

**Figure 3:**
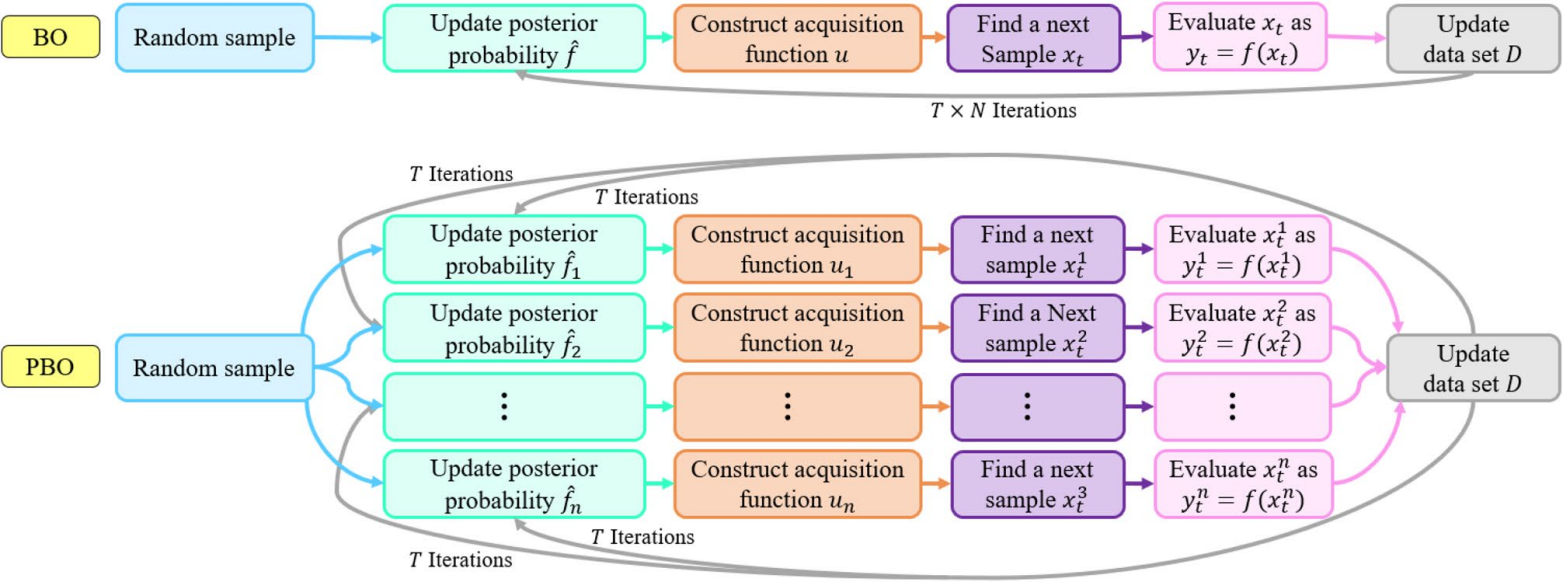
Algorithmic diagrams for PBO and BO.

We develop a new parallel optimization scheme for the optimization part of the time-dependent parameter calibration framework, which addresses the BO method’s drawbacks. In this approach, instead of allocating all searching budget (the number of iteration) to one BO model, we divide the searching budget into several BO algorithms to search a solution space in parallel. To address BO’s sensitivity to the type of acquisition functions, each parallel BO has a specific type of acquisition function. Besides, each BO applies a particular kernel type and hyper-parameters for constructing the GP distribution. When BO models determine the next sample point by maximizing its equations function, they share their findings to help each other to acquire more knowledge about the solution space. The structural differences between BO and PBO are illustrated in Fig. 3. The PBO algorithm is defined in Algorithm 1.

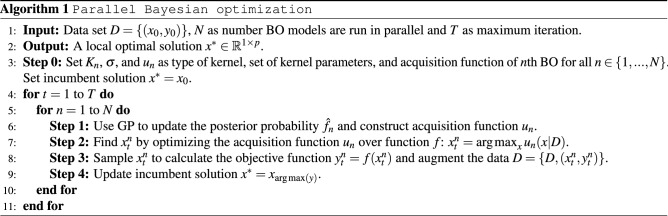



We provide an additional explanation about the algorithm as follows.

- *Hyperparameters* Parameter *N* should reflect the number of parallel BO models such that any increase in *N* will greatly expand the searching power to search through more enumeration. The maximum number of iterations *T* indicates the stop criteria of the algorithm. The maximum number of iterations can be replaced with a threshold so that we terminate the algorithm if the difference between the current value of the best new points and the incumbent solution is less than a predefined threshold.
- *Step 0* In this step, we set one type of acquisition function, kernel, and kernel parameters for constructing the GP of each BO model.
- *Step 1* GP updates the posterior probability, and constructs acquisition function in this step.
- *Step 2* We use the limited-memory quasi-Newton algorithm for bound-constrained optimization $\text {L-BFGS-B}$^69,70^ in Python to optimize the acquisition function with a set of random starting points. We run $\text {L-BFGS-B}$ several times with different arbitrary starting points to improve the efficiency and overcome the difficulty of optimizing the acquisition function. We select the next sample point as the best optimal solution between all found solutions of $\text {L-BFGS-B}$ algorithm with different starting points.
- *Step 3* The APSIM is run to evaluate a new parameter combination from each BO model in this step. Then, the BO models share their findings to expand their knowledge about posterior distribution such that the BO model applies GP in the next iteration to update its posterior distribution and construct the acquisition function.
- *Step 4* The incumbent solution is updated as the best combinations of parameters evaluated by APSIM in this step.

## Case study

**Figure 4:**
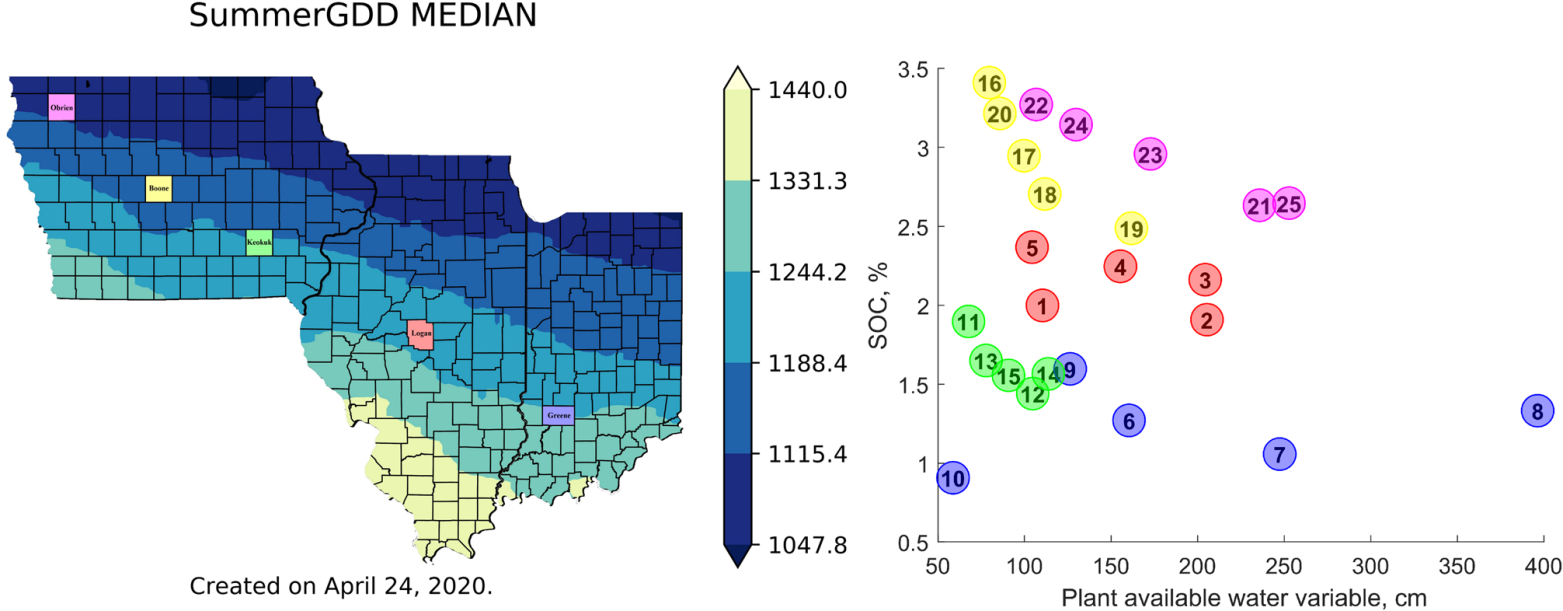
(Left) 34-year average thermal time sum ($^{\circ }$C-days) from June 1 to August 31 for 3 states in the US Corn Belt. The five counties used to test the framework are shown on the map. (Right) The plot shows the scattering of selected locations in terms of soil organic carbon (in %) and plant available water (in cm). The circles with the same color illustrate locations in one specific county. The cultivars maturity in locations #1 to #5 are 114-day hybrids, locations #6 to #10 are 115-day hybrids, location #11 to #15 are 112-day hybrids, for locations #16 to #20 are 110-day hybrids, locations #21 to #22 are 107-day hybrids, and locations #23 to #25 are 108-day hybrids.

A case study with 25 locations from 1984 to 2019 (34 years) has been designed to illustrate how good the proposed time-dependent parameter estimation method is. We used the APSIM maize model^71^ (version 7.9), and in particular, the APSIM simulation files outlined by Archontoulis et al.^14^ that comprise simulation of shallow water tables and inhibition of root growth due to excess water stress^18^, and waterlogging functions^72^. We used APSIM because it is open-source software, advanced simulator of the cropping systems, and the model is commonly used in the US Corn Belt as well as worldwide^73^. We selected 5 counties (Logan (IL), Greene (IN), Keokuk (IA), Boone (IA), and Obrien (IA)) across the US Corn Belt and within each county 5 different soils, generating a range of variability in the weather and soil inputs (see Fig. 4). The APSIM simulation files were generated using pSIMS^74^, which is used to run APSIM across the US Corn Belt at a resolution of 5 arcminutes^19,75^. Each APSIM simulation file contained a soil profile, a weather file, the historical management, including N-fertilization, planting dates, plant population per year and state, and the simulated yield by year as well as a number of other soil-plant processes. Historical statewide plant density and planting dates per year were derived from USDA NASS quick stats^76^. County-level N fertilization rate was derived from a combined analysis of Cao et al.^77^ and USDA NASS^76^. The same observed yield for five locations at each county is used because the county-level yield data was acquired from the National Agricultural Statistics Service of the United States^76^. Since the management and the environment (weather $\times$ soil) is already captured in the model, the only unknown is the cultivar parameters and how these change from year to year or from decade to decade. Climate information derived from a reanalysis weather database based on NASA Power^79^ and Iowa Environmental Mesonet^80^, both daily historical weather sources.

For each county, we used a K-medoids method^81^ as a clustering technique to select the five locations with the most distinct soil information. Figure 4 (right) shows the variability in soil organic carbon (in %) and plant available water (the difference between field capacity and wilting point in cm; depth 0–150 cm) across the 25 soils. We used adapted cultivars within a county in terms of relative maturities, given the 1.5-fold gradient in temperature from southeast to northwest (see Fig. 4).

### Experiment settings

In this study, we considered the prediction of corn yield increase from 1985 to 2018 to evaluate the performance of the developed framework in estimating nine time-dependent parameters that can capture the observed historical yield increase: 8160 kg/ha to 12,679 kg/ha. We selected the following parameters summarized in Table 1 to optimize for two reasons: (1) their known sensitivity on yield and (2) evidence that these parameters have changed with plant breeding from 1980 to today^51–56^.

**Table 1:** List of parameters and acronyms used in this study, with their definitions, values, and units.

Parameter or abbreviation	Definition	Value or Range	Unit
tt_emerg_to_endjuv	Thermal time from emergence to end of juvenile phase	[150,300]	$^{\circ }$Cd
tt_flower_to_maturity	Thermal time from flowering to maturity	[600,900]	$^{\circ }$Cd
head_grain_no_max	Potential grains per head	[600,850]	kernel/ear
grain_gth_rate	Potential grain growth rate	[5,9]	mg/grain/d
tt_flower_to_start_grain	Thermal time from flowering to start grain fill	[120,200]	$^{\circ }$Cd
n_conc_crit_grain	Critical N concentration of grain	[0.008,0.016]	g N/g biomass
leaf_app_rate1	Thermal time required to develop a leaf ligule for first leaves	[50,75]	deg day
Rue (max_stage)	Radiation use efficiency in each stage	[1.4,1.85]	g dm/mj
transp_eff_cf	Transpiration efficiency coefficient	[0.075,0.095]	kpa

This case study was conducted for two purposes. First, to test the proposed optimization method in the proposed framework, we calibrate the time-dependent APSIM parameters to predict historical corn yield increase with manual calibration, the proposed framework using BO and PBO algorithms. In the manual calibration, the following six parameters were changed every 5-years in the model^51^: potential kernel number ear, grain growth rate, thermal time from silking to start grain filling, the radiation use efficiency, the transpiration use efficiency, and finally the grain nitrogen critical concentration. The direction and magnitude of changes were informed from literature studies^51–56^. Simultaneously, the results were used to benchmark new predictions using the crop model with tuned parameters from the proposed framework using BO and PBO algorithms. For the APSIM-BO and APSIM-PBO calibration, we follow a similar approach, but we allowed three additional parameters to change over time for a total of 9 parameters (Table 1). The 1985 to 2018 period was divided into 30 time-windows such that each main time-window contains five years. The proposed framework was implemented in Python 3.

Second, we benchmarked the yield prediction performance of the proposed parameter calibration method with machine learning models to illustrate the impact of the proposed time-dependent parameter estimation framework in enhancing the crop model’s performance. Nine machine learning models applied for this benchmark are summarized as follows:

- Linear regression I model was implemented in Python using the Sklearn package^82^.
- Linear regression II model was implemented in Python using the Sklearn package^82^.
- Lasso model was trained using the Sklearn package^82^ in Python.
- Ridge regression was implemented using the Sklearn package^82^ in Python.
- Elastic net was trained using the Sklearn package^82^ in Python. Its hyper-parameter “alpha” was tuned using five-fold cross-validation (without data leakage).
- Bayesian ridge was implemented in Python using the Sklearn package^82^. Hyper-parameters lambda (precision of the weights) and alpha (precision of the noise) were using five-fold cross-validation (without data leakage).
- Gradient boosting was trained using the Sklearn package^82^ in Python, which was an efficient and scalable implementation of gradient boosting framework. Three hyper-parameters were tuned using five-fold cross-validation (without data leakage): “max_depth” (depth of tree), “n_estimators” (number of trees), and “max_features” (number of features).
- Random forest was trained using the Sklearn package^82^ in Python, which was an ensemble of decision trees and trains with the bagging method. Three hyper-parameters were tuned using five-fold cross-validation: “n_estimators” (number of trees), “max_depth” (depth of tree), and “max_features” (number of features).
- Deep neural network was implemented in Python using the Sklearn package^82^. Hidden layer sizes and the number of hidden layers as its hype-parameter were tuned using five-fold cross-validation.

For this test, we considered the last five years. To predict corn yield at test year $Y+1$ with APSIM crop model, the historical data set, including all the environment (weather and soil), management, and crop information, and crop yield data from 1985 to year *Y* are applied to tune the parameters using the proposed framework. After fitting the autoregressive model to historical tuned parameters and estimating the parameter values for test year $Y+1$, we ran APSIM with the estimated parameters at year $Y+1$ to predict the corn yield.

To train the machine learning models, we create data set with soil profiles from SSURGO^78^, climate information from NASA Power^79^ and Iowa Environmental Mesonet^80^, and historical management information and yield performance data^76^ for all 293 counties of the states of Illinois, Indiana, and Iowa from 1985 to 2018. Weather data included four weekly average surface weather parameters (converted from daily data): precipitation (mm), solar radiation (MJ/m$^2$), maximum temperature (C$^\circ$), and minimum temperature (C$^\circ$) from weeks 13 (late March) to 52 (late December). Soil variables included dry bulk density (g cm$^{-3}$), clay percentage (%), soil pH, drained upper limit (mm mm$^{-1}$), saturated hydraulic conductivity (mm/day), drained lower limit (mm mm$^{-1}$), organic matter (%), sand percentage (%), and saturation (mm mm$^{-1}$) at nine different depths of soil: 0–5, 5–10, 10–15, 15–30, 30–45, 45–60, 60–80, 80–100, and 100–120 cm. Management variables included acres planted at the county level, weekly cumulative percentage of planted and harvested acreages, and nitrogen applications at the county level. Therefore, the total number of variables is 324 (including 160 for weather, 81 for soil, 83 for management).

The time variable included the year of observation to capture the changes in crop parameter values over the years. The created data set was divided into training and test data sets. The training data set contains the historical information from 1985 to year *Y* for all 293 counties, and the test data set is comprised of information of year $Y+1$ for five considered counties. We used 293 counties so that the machine learning models have enough data set to train and figure out the relationship between input and response variables. We tuned the machine learning models’ hyper-parameters and trained them using the training data set, and then their performances were evaluated using the test data set. The first linear regression model (Linear regression I) fits the linear line on county-yield over time such that it represents a linear trend of county-yield vs time. However, other machine learning models intend to predict county-yield using weather, soil, management and time information. A feature selection step was performed to select a set of high-quality features before fitting the data set to machine learning models. We applied forward and backward stepwise selection by considering a 10-fold cross-validation schema to identify features.

Three criteria were used for comparing and evaluating the prediction performance of models: root mean square error (RMSE), which computes the difference between predicted and observed yield, relative RMSE (RRMSE), which measures the normalized difference between predicted and observed yield, and coefficient of determination ($R^2$), which calculates the proportion of the variance in the yield that is explained by independent variables.

The proposed framework was implemented in Python 3. We performed the experiments on a workstation with a 3.4 GHz CPU and 16 GB memory. The training was done by location such that calibration of these nine parameters at each time-window for one location took 8 CPU hours. The workstation’s CPU allows us to run 6 combinations of locations by time-windows at the same time. Hence, the total computation time for estimating nine parameters in all combinations of location by time-window (30 $\times$ 25) was approximately 41 CPU days.

## Results

### Comparison with other parameter calibration methods

To compare with other parameter calibration methods, the median predicted yield of five locations in each county is used instead of the mean because it reduces the outliers’ effect on county-level yield. The result shows that the proposed framework with the PBO algorithm outperformed the BO and the manual approaches in terms of yield simulation from 1985 to 2018 (Table 2). Compared to the manual optimizer, the PBO optimizer decreased the RMSE by 47% at Logan and up to 65% at Obrien. The PBO optimizer has also achieved lower error than the BO optimizer, at least 4% at Greene and up to 16% at Boone. This performance can be attributed to using the proposed optimization algorithm in parameter tuning.

**Table 2:** RMSE in kg/ha (and RRMSE) for five selected counties over simulation period (1985 to 2018).

Method	Logan (IL)	Greene (IN)	Keokuk (IA)	Boone (IA)	Obrien (IA)
APSIM-Manual	2386 (23.03%)	2820 (33.48%)	2612 (29.36%)	2357 (23.69%)	2482 (24.42%)
APSIM-BO	1442 (13.93%)	1520 (18.05%)	1590 (17.88%)	1288 (12.95%)	992 (9.77%)
APSIM-PBO	1266 (12.23%)	1459 (17.32%)	1358 (15.26%)	1076 (10.82%)	865 (8.51%)

**Figure 5:**
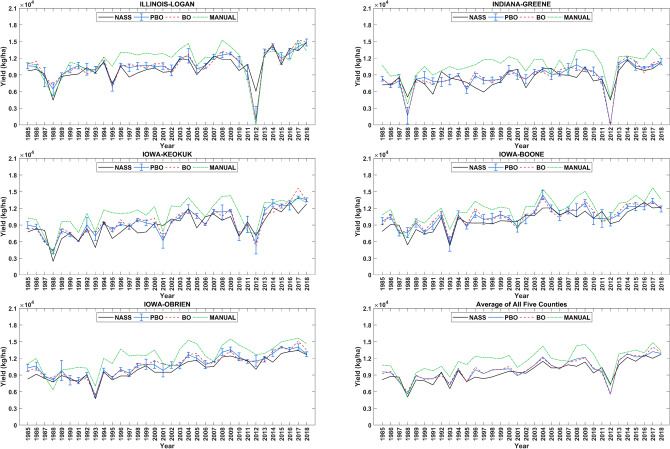
Yield predictions of five selected counties for the simulation period (1985–2018). Five sub-figure corresponds to five counties, and one indicates the average of all five counties. The black line indicates the observed county-level yield from the National Agricultural Statistics Service of the United States. For each county, the median predicted yields of five locations from PBO, BO, and manual methods are shown by a solid blue line, dashed red line, and dash-dot green line, respectively. The error bar illustrates our prediction interval that shows the standard deviation of prediction over five locations at each year.

Figure 5 shows the time-series simulations. The median predicted yield of five locations at each county was considered as the predicted yield of counties because of county-level observed yield. Each sub-figure corresponds to one county in this figure, and the error bar illustrates our prediction interval at each year. According to the result, the consistency of the simulated yield performance across the corn belt’s spatial and temporal dimensions indicates the proposed framework’s effectiveness using PBO optimizer in the calibration of time-dependent parameters in APSIM. Another observation is that the proposed framework can determine the time-dependent parameters’ values to capture both the overall increasing trend of yield over simulation years and yield fluctuations from one year to another year.

### Prediction accuracy comparison with other machine learning models

Prediction performance of five test years using the crop model with estimated cultivar parameters and machine learning models are reported in Table 3. These results indicate that the proposed framework with the PBO method outperformed other approaches for four out of five test years. It seems that close-to-optimal parameters’ values from the proposed framework help APSIM to simulate the yield more accurately and significantly reduce the crop yield prediction error.

The goodness of fit of the proposed framework with the different optimizer and random forest in crop yield prediction problem suggest that the prediction of APSIM with parameters from the proposed time-dependent parameter is close to the observation (the better distribution of the residuals derived from the distance between predicted and observed yield corresponding to line $x=y$), which decreased overall prediction bias.

**Table 3:** Prediction performance of the proposed approach and 8 other approaches for five selected counties for five test years (2014 to 2018) at the end of the growing season.

Criteria	Method	Test year				
		2014	2015	2016	2017	2018
RMSE	Linear regression I	2170	738	1567	742	1463
	Linear regression II	1734	1476	1723	1110	1405
	Lasso	1143	927	999	834	657
	Ridge	1153	2137	1565	1427	1123
	Elastic net	1214	1430	1848	979	997
	Bayesian ridge	1153	1099	1382	758	698
	Gradient boosting	944	840	1162	713	359
	Random forest	910	547	1103	**593**	430
	Deep neural network	923	705	992	717	493
	APSIM-Manual	1549	2041	1623	2926	1337
	APSIM-BO	955	631	904	685	498
	APSIM-PBO	**832**	**518**	**818**	630	**293**
RRMSE	Linear regression I	17.80%	6.43%	12.45%	6.18%	11.48%
	Linear regression II	14.22%	12.86%	13.68%	9.24%	11.03%
	Lasso	9.38%	8.08%	7.94%	6.94%	5.16%
	Ridge	9.45%	18.62%	12.43%	11.88%	8.82%
	Elastic net	9.96%	12.46%	14.67%	8.15%	7.83%
	Bayesian ridge	9.46%	9.58%	10.98%	6.31%	5.48%
	Gradient boosting	7.74%	7.32%	9.22%	5.94%	2.82%
	Random forest	7.46%	4.77%	8.76%	**4.94%**	3.38%
	Deep neural network	7.57%	6.14%	7.87%	5.97%	3.87%
	APSIM-Manual	12.70%	17.79%	12.89%	24.36%	10.50%
	APSIM-BO	7.84%	5.50%	7.18%	5.71%	3.91%
	APSIM-PBO	**6.82%**	**4.51%**	**6.50%**	5.25%	**2.30%**
R2	Linear regression I	-2.36	0.36	-0.21	0.66	-0.53
	Linear regression II	0.03	-0.24	-0.42	0.75	0.37
	Lasso	0.09	0.11	0.63	0.60	0.69
	Ridge	0.43	-0.20	-0.19	0.71	0.40
	Elastic net	0.42	0.01	-0.57	0.81	0.55
	Bayesian ridge	0.30	0.00	0.05	0.78	0.70
	Gradient boosting	0.47	0.31	0.36	0.81	0.91
	Random forest	0.46	0.66	0.42	**0.84**	0.87
	Deep neural network	0.39	0.43	0.59	0.73	0.82
	APSIM-Manual	-0.05	0.16	-0.19	0.11	-0.02
	APSIM-BO	0.37	0.56	0.69	0.75	0.84
	APSIM-PBO	**0.50**	**0.69**	**0.72**	0.79	**0.93**

Bold values are used to highlight the result of the best models.

### Time-dependent parameter estimates

**Figure 6:**
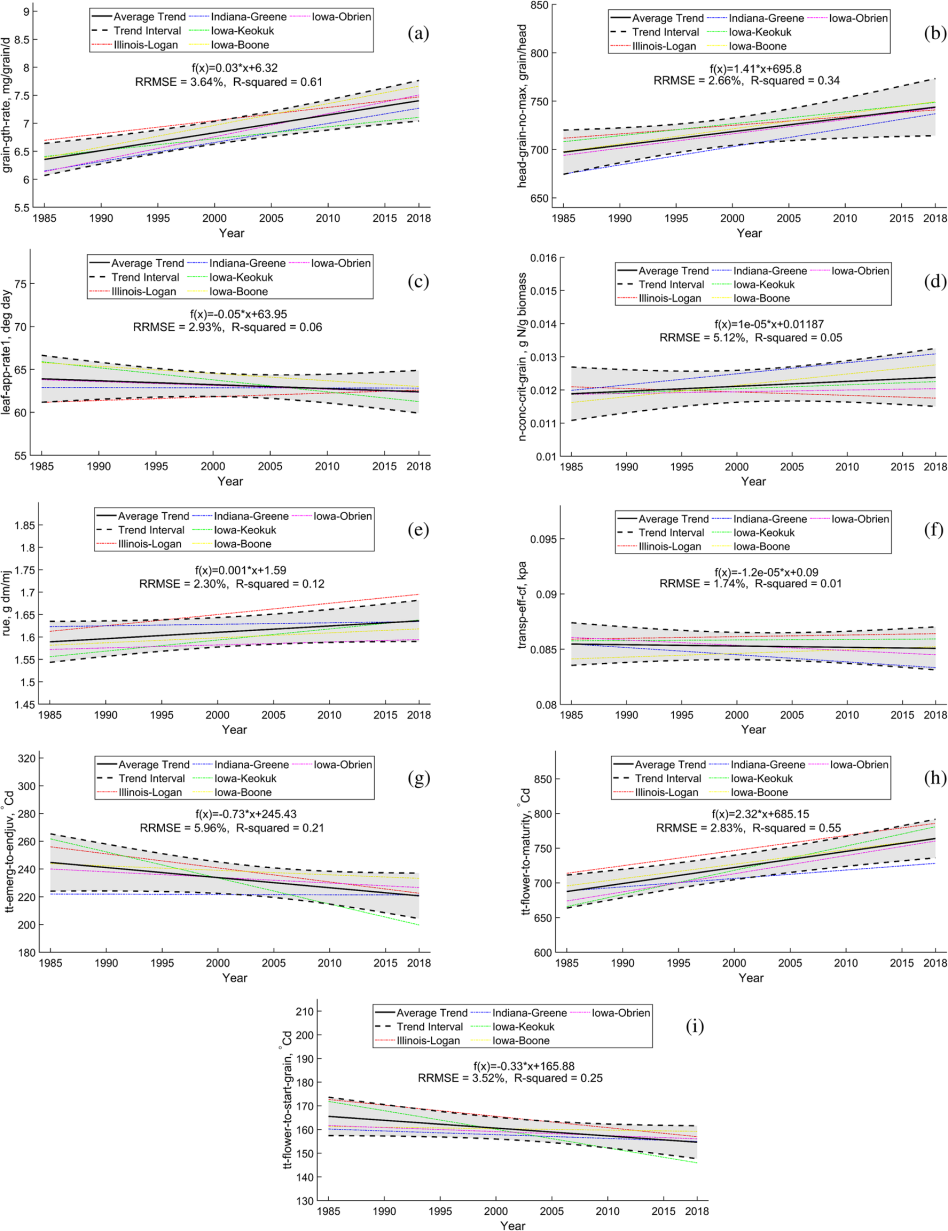
Each subplot indicates the linear trend of each county’s tuned parameter and its interval value during the simulation period (1985–2018). For each county, the average trend of its locations is shown with the dash-dot line. The dash-dot colored lines show the linear trend of tuned parameters at different counties. The solid black lines and dashed black lines illustrate the average ± standard deviation of linear parameters’ trends over all five counties.

The linear trend of tuned parameters for five counties over time is illustrated in Fig. 6 with five dash-dot colored lines such that each color corresponds to a specific county. For each county, the average trend its locations is shown with the dash-dot line. The solid black lines and dashed black lines indicate average and standard deviation of parameters’ trends over five counties. The confidence interval indicates the range of variability across 25 environments. We did not find any correlation between optimized parameters and soil or weather variables to explain this range of variability. Probably, it is the result of a complex interaction. The slope of the linear trend of parameters is reported in Table 4.

**Table 4:** The linear trends of estimated parameters with the proposed parameter calibration framework with PBO method.

Parameter or abbreviation	Measurement	Change per year
tt_emerg_to_endjuv	Determines silking time	$-\,0.73$ $^{\circ }$Cd
tt_flower_to_maturity	Determines the silking to maturity duration	2.32 $^{\circ }$Cd
head_grain_no_max	Determines the potential number of kernels per ear	1.41 kernel/ear
grain_gth_rate	Determines the grain growth rate (proxy for harvest index)	0.03 mg/grain/d
tt_flower_to_start_grain	Determines the duration from silking to start grain filling	$-0.33$ $^{\circ }$Cd
n_conc_crit_grain	Determines the critical grain N concentration	$1 \times 10^{-5}$ g N/g biomass
leaf_app_rate1	Determines the how fast the first 10 leaves appear	$-0.05$ deg day
Rue (max_stage)	Reflects the canopy photosynthetic capacity	0.001 g dm/mj
transp_eff_cf	Determines the efficiency of water	$-1.2\times 10^{-5}$ kpa

The PBO estimated parameter values indicated that the RUE parameter increases linearly from 1985 to 2018 with an average slope of 0.001 g dm/mj. The transpiration efficiency parameter remained the same. Both grain component parameters (grain growth rate and potential kernel number per ear) increased over time with an average slope of 0.03 mg/grain/d and 1.41 kernel/ear, respectively. The leaf appearance rate decreased over time with an average slope of 0.05 deg day, indicating lower values for this parameter and thus faster leaf production. Among three thermal time parameters explored in this study, the emergence to a juvenile parameter that refers to the vegetative phase duration was decreased by 0.73 $^{\circ }$Cd per year, the flowering to the maturity parameter that refers to the reproductive phase duration was increased by 2.32 $^{\circ }$Cd per year, while the flowering to start seed parameter decreased by 0.33 $^{\circ }$Cd per year. Finally, the parameter related to grain protein (n_conc_crit_grain) was very slightly increased. Also, in terms of the estimated parameters’ variation range over the 25 environments, the largest variation was observed for the tt_emerg_to_endjuv parameter.

## Discussion

This study developed and demonstrated a new time-dependent parameter estimation framework for the APSIM crop model that captures well historical yield increase in the US Corn Belt. To the best of our knowledge, this is the first study that develops a new automated framework to estimate time-dependent cultivar related parameters. Compared to other calibration methods (manual^17^ and BO^39,45^ methods) in the literature, our models can capture the trend of the crop model’s output by determining the temporal dependency of parameters. While the proposed framework was designed to enhance the crop model’s performance in simulating nature by calibrating its model parameters, we demonstrated its effectiveness by comparing its prediction accuracy of crop with that of machine learning models, which were specifically trained for prediction accuracy and not for parameter calibration. Results from our case study showed that the prediction accuracy of the calibrated crop model with the proposed framework was favorably comparable with that of machine learning models as well as what has been reported in the literature of crop yield prediction^75,83–86^.

Table 2 and Fig. 5 indicate that APSIM with estimated parameters of the proposed approach with the PBO optimizer was able to capture observed yield trend and yield fluctuations from one year to another year during the simulation period. The improved prediction performance was also more generalizable to the test data set, as demonstrated in Table 3. The proposed framework’s revealed trends of plant traits that have changed from 1985 to 2015 over the year due to crop improvement. Furthermore, results from this study can stimulate similar cultivar parameter calibration efforts in other crop models used for yield prediction and climate change assessments in the US Corn Belt and beyond. Our study offers an alternative way to the traditional field experimentation, era studies, to understand the reasons for the historical yield increase in the US Corn Belt, although ultimate validation of key parameters will still require field experiments.

Although we used the autoregressive model to capture the time-dependent trend of parameters over time in the proposed framework, we can use a linear regression model instead of an autoregressive model to capture the linear parameters’ trend from 1985 to 2018. More details about the autoregressive model’s performance on estimating weighted average parameters values in the proposed framework are provided in Appendix 2.

The estimated trend in RUE parameter increasing from 1985 to 2018 is consistent with era studies, in which cultivars from different eras were compared^51,52^. In these studies, the authors estimated an RUE increase of $0.0057 \pm 0.002$ g dm/mj per year , which is close to our estimate. The PBO approach indicated that the transpiration efficiency coefficient did not change over time, however, as it is shown in Fig. 6f there was quite some variation among the 25 environments, with some environment to show an increase. Literature studies^52,87,88^ have indicated an increase in water use efficiency, a proxy for the transpiration use efficiency parameter. Perhaps the disagreement between our estimate and literature estimates comes from the soil that used (e.g. soil with or without watertable) and the overall simulation of the water balance.

Regarding the grain components, the PBO indicated that both the grain growth rate (a proxy for the harvest index) and the potential kernel number have increased over time, which in general follows literature experimental findings^52,55,56,89–92^. For example, the kernels per ear parameter in our study increased from 700 to 750, while the range of increase that has been reported in the literature using different era hybrids and different environments ranges^55^. The estimated increase in grain filling period by about 100 $^{\circ }$Cd ($\sim 5$ days) over the years agrees with experimental reports^53,90^, satellite data analysis^93,94^, and NASA phenological data analysis^95^. We did not find relevant literature to benchmark our estimates for the other three phenological parameters (leaf appearance rate, vegetative stage duration, and thermal from flowering to start grain filling).

In contrast to previous studies^54,56,92,96^ that suggest a decline a grain protein (and therefore grain N concentration) with years of hybrid release, the estimated critical grain N concentration parameter by the PBO approach did not show a consistent decreasing trend over time (e.g., Figs. 5, 18, and 21 in Appendix 2). In 14 of the 25 cases (5 soils × 5 counties), the critical grain N concentration increased over time (linear slope is more than $1 \times 10^{-5}$ g N/g biomass per year), in 6 out of the 25 cases decreased over time (linear slope is less than $-1 \times 10^{-5}$ g N/g biomass per year) while in 5 out of the 25 cases remained unchanged over time (linear slope is between $-1 \times 10^{-5}$ g N/g biomass per year and $1 \times 10^{-5}$ g N/g biomass per year) (e.g., Figs. 8 and 17 in Appendix 2). As the only one out of nine calibrated parameters that showed an inconsistent trend with the literature (as well as manual APSIM calibration), this may be an artifact of “equifinality”, i.e., different combinations of parameter values can yield the same result^20,97,98^ in the PBO approach, which suggest the need for a) more data on grain N concentration to better estimate this parameter or b) development of alternative algorithms. There may also be inconsistency in its definition as the critical grain N concentration parameter in the crop model sets a target, which influences the final grain N concentration estimation, but it is not equivalent to the final grain N concentration that is measured in field experiments. The grain N concentration is a very complex trait. Many other processes influence its final value, including soil N mineralization, N leaching, denitrification, crop N uptake, and fertilizer input in addition to the critical grain N concentration parameter.

Despite its encouraging performance, the proposed approach has its limitations. While yield variation can be influenced by a large number of parameters in APSIM, we chose the nine parameters we considered most likely to vary in the Midwestern germplasm as free parameters. The possibility remains that there are alternative combinations of traits that underlie the yield changes between 1985 and 2018, which potentially explains why fitted values like critical grain N concentration are not consistent with experimental observations. Choosing alternative combinations of free parameters may suggest alternative explanations for yield changes.

A recent study^99^ reported that county-level yield data are insufficient to test crop model performance in extreme weather years (e.g., the year 2012). Our simulations did not provide evidence for this. This is because our model setup included shallow water tables that may have positive and negative impacts on productivity^14^, especially in extreme weather years such as 2012. We used the autoregressive model to capture the parameters’ trends; the modeling framework in Fig. 2 was based on this time-series model. To accommodate other trends, one can replace the autoregressive model with different time series models. Moreover, the PBO algorithm is computationally complex and sensitive to a number of parameters. In our case study, only nine parameters needed to be calibrated; when this number increases to many dozens, the computational time for the proposed framework could significantly increase.

Beyond parameter calibration for APSIM, the new approach can be used for a wide variety of studies that involve parameter calibration, especially those whose parameters demonstrate specific patterns over time. The modeling framework illustrated in Fig. 2 can be readily modified to accommodate other models. Future research should further explore the environmental and management parameters to make more biologically and agronomically insightful discoveries. Moreover, this framework can be used to optimize both short-term and long-term farm management strategies.

## Conclusions

We developed and demonstrated a new framework to estimate time-dependent parameters’ values for crop models to improve performance with regards to capturing historical yield trends. We integrated the power of optimization, machine learning, and prior agronomic knowledge to develop a new automated parameter calibration algorithm. The developed PBO algorithm search for the best combination of parameters in high dimensional space to enhance the exploration and exploitation of the optimization step in the proposed framework. A computational case study using 25 environments in the US Corn Belt, each with 34 years of data, confirmed that the proposed framework outperformed the state-of-the-art calibration model (BO and manual calibration) and enhanced crop model performance in corn yield prediction accuracy so that it can outperform accurate machine learning models in the literature. Finally, this work produced insights about plant traits’ changes over the last 34 years, offering an alternative way to deeper understand historical yield increases in the US Corn Belt and inform future experimentation.

## Supplementary Information

Supplementary Information.
