# Supplementary material for: A time-dependent parameter estimation framework for crop modeling
Source: Sci Rep. 2021 Jun 1;11:11437. doi: 10.1038/s41598-021-90835-x (PMC8169860; doi:10.1038/s41598-021-90835-x)
Supplement: Supplementary file 1 — Supplementary Information. [file 41598_2021_90835_MOESM1_ESM.pdf]

Developing Time-dependent Parameters Estimation Framework for Crop Modeling

Faezeh Akhavizadegan[1,*], Javad Ansarifar[1], Lizhi Wang[1], Isaiah Huber[2], and Sotirios V. Archontoulis[2]

[1]Department of Industrial and Manufacturing Systems Engineering, Iowa State University, Ames, IA 50011, USA
[2]Department of Agronomy, Iowa State University, Ames, IA 50011, USA
[*]Faezeh.akhavizadegan@gmail.com

Appendix 1

Bayesian Optimization

Like other kinds of optimization methods, BO is a framework to find the maximum value for the following problem with unknown function $f : x^* = \arg\max_{x \in A} f(x)$ where A denotes the search space of x. BO is a very effective method for solving optimization framework assuming that evaluating the function $f(x)$ is computationally expensive or does not have a closed-form expression. What makes BO different from other optimization methods is the BO optimizes a random function $f(x)$ without the requirement of an explicit expression of the function $f(x)$. The essential philosophy of BO is construction of a probabilistic model for $f(x)$.

Bayes' theorem[1] is used to consider the uncertainty in the estimation of $f(x)$. BO uses posterior information of function $f(x)$ for maximization, where the posterior information is acquired by combining its prior distribution with the sample information (evidence). The prior distribution is not an objective basis. It is often based on subjective beliefs, where BO assumes a prior distribution of $f(x)$ as a Gaussian process, which is well-suited prior distribution and is highly flexible and easy to handle[1].

Because of the non-convexity nature of function $f(x)$, evaluating and optimizing $f(x)$ is non-trivial. Therefore, methods for finding the optimal solution are point-based evaluations and not gradient or Hessian based evaluations. Optimizing within the search space A needs to take into account both exploration (search new sample in the space with high uncertainty) and exploitation (searching new sample in the space with high expected values)[2]. This trade-off between exploration and exploitation helps to decrease the number of point evaluations and improve the performance of BO. BO maximizes the function $f(x)$ by maximizing acquisition function so that the large value of the acquisition function u corresponds to the high value of the function f. This acquisition function is a trade-off between exploration and exploitation during the determination of the next sample point to maximize the acquisition function.

Algorithm 1 shows the BO structure as an iterative approach. The idea of classic BO is composed of two parts. First, the posterior distribution was updated using GP. Second, the next point is determined by maximizing the acquisition function. BO starts with random solution (x_0, y_0). At iteration t, GP updates the posterior distribution of $f(x)$ to develop acquisition function using observed sample information D. Then, the acquisition function tries to find the next point x_t corresponding to the maximization of the acquisition function. After that, the new point x_t is evaluated to calculate real value $f(x_t)$ as y_t in step 3. Then, we augment observed sample information D with a new sample. GP updates the posterior distribution at the next iteration. The whole procedure is continued until the maximum number of iterations T is reached.

Algorithm 1 `Bayesian optimization`

Input: Data set $D = \{(x_0, y_0)\}$. T as maximum number of iterations.
Output: A local optimal solution $x^* \in \mathbb{R}^{1\times p}$.
Step 0: Set incumbent solution $x^* = x_0$. Set K, σ, and u as type of kernel, set of kernel parameters, and acquisition function.

for $t = 1$ to T **do**
  Step 1: Use GP to update the posterior probability $\hat{f}$ and construct acquisition function u.
  Step 2: Find x_t by optimizing the acquisition function u over function f: $x_t = \arg\max_x u(x|D)$.
  Step 3: Sample x_t to calculate the objective function $y_t = f(x_t)$ and augment the data $D = \{D, (x_t, y_t)\}$.
  Step 4: Update incumbent solution $x^* = x_{\arg\max(y)}$.
end for

Gaussian Processes

GP as a stochastic process is a generalization of the Gaussian distribution such that any finite subset of which has a multivariate Gaussian distribution[3]. Distribution over functions $f(.) \sim GP(\mu(.), k(.,.))$ is Gaussian Process, where mean function $\mu(.)$ and covariance kernel $k(x,x')$ for any pairs of input points $x,x' \in \mathbb{R}^{1\times p}$ are defined as follows,

$$\mu(x) = \mathbb{E}[f(x)], \qquad k(x,x') = \mathbb{E}[f(x)-\mu(x))(f(x')-\mu(x')].$$

For a given finite set of input points ($X = \{x_1, x_2, ..., x_n\}$, $f(X) = \{f(x_1), f(x_2), ..., f(x_n)\}$), their associated random variables have distribution

$$\begin{bmatrix} f(x_1) \\ \vdots \\ f(x_n) \end{bmatrix} \sim N\left(\begin{bmatrix} \mu(x_1) \\ \vdots \\ \mu(x_n) \end{bmatrix}, \begin{bmatrix} k(x_1,x_1) & \cdots & k(x_1,x_n) \\ \vdots & \ddots & \vdots \\ k(x_n,x_1) & \cdots & k(x_n,x_n) \end{bmatrix} \right).$$

For convenience, BO assumes the zero-mean Gaussian process ($\mu(.) = 0$), and the GP's power to fit posterior distribution relies on the shoulders of the covariance function. For the covariance function k, various types of kernels have been defined. The most popular ones are:

$$\textbf{Squared Exponential Kernel: } k(x,x') = \sigma_f^2 exp(-\frac{1}{2}\frac{||x-x'||^2}{\sigma_l^2})$$

$$\textbf{Exponential Kernel: } k(x,x') = \sigma_f^2 exp(-\frac{||x-x'||}{\sigma_l^2})$$

$$\textbf{Matern 3/2: } k(x,x') = \sigma_f^2(1+\frac{\sqrt{3}||x-x'||}{\sigma_l})exp(-\frac{\sqrt{3}||x-x'||}{\sigma_l})$$

$$\textbf{Matern 5/2: } k(x,x') = \sigma_f^2(1+\frac{\sqrt{5}||x-x'||}{\sigma_l}+\frac{\sqrt{5}||x-x'||^2}{\sigma_l^2})exp(-\frac{\sqrt{5}||x-x'||}{\sigma_l}),$$

where σ_f is a signal standard deviation parameter, σ_l is a characteristic length scale parameter and $x,x' \in \mathbb{R}^{1\times p}$ are pair of input points. At each iteration, BO uses the observed samples $D = \{(X, f(X))\}$ to fit posterior probabilities for new query point $\hat{x}$ by applying GP. A new query point and observed samples have the same Gaussian distribution as follows,

$$\begin{bmatrix} f(X) \\ f(\hat{x}) \end{bmatrix} \sim N\left(\begin{bmatrix} \mu(X) \\ \mu(\hat{x}) \end{bmatrix}, \begin{bmatrix} k(X,X) & k(X,\hat{x}) \\ k(\hat{x},X) & k(\hat{x},\hat{x}) \end{bmatrix} \right).$$

By applying Bayes's rule to compute the posterior probability, we have $\hat{f}(\hat{x}) \sim Pr(f(\hat{x})|\hat{x},(X,f(X))) = N(\mu_f(\hat{x}), \Sigma_f(\hat{x}))$, where $\mu_f(\hat{x}) = \mu(\hat{x}) + k(\hat{x},X)k(X,X)^{-1}(f(X)-\mu(X)), \Sigma_f(\hat{x}) = k(\hat{x},\hat{x}) - k(\hat{x},X)k(X,X)^{-1}k(X,\hat{x})$. Instead of the scalar value for new query point $\hat{x}$, GP returns the probability distribution over all possible values of $f(\hat{x})$ such that if the D is large enough, GP can provide a close estimation of the function $f(\hat{x})$ distribution.

Acquisition Functions

After construction of posterior distribution of the function $f(x)$, BO tries to find the new test point $\hat{x}$ in order to maximize the function f. For this purpose, BO utilizes the acquisition function u to determine the next test point $\hat{x}$ by considering the uncertainty in posterior distribution and trade-off between exploration and exploitation. Maximizing the acquisition function is equivalent to maximizing the function f. In general, the acquisition function u relies on the previous sample observations and GP hyperparameters. Several acquisition functions have been defined as follows:

Probability of Improvement as an intuitive utility function that maximizes the probability of improving over the incumbent solution x^*[4] is defined as follows,

$$\text{PI}(\hat{x}) = Pr(f(\hat{x}) \geq f(x^*)) = \phi(\frac{\mu_f(\hat{x}) - f(x^*)}{\Sigma_f(\hat{x})}),$$

where $\phi(.)$ is the cumulative distribution function of the standard normal distribution. The idea of this acquisition function is intuitive because it takes sample $\hat{x}$ from the areas near the incumbent solution x^* which is the optimal solution of exiting acquisition function. Therefore, as one of its drawbacks, it focuses on the exploitation and takes the sample in limited ranges, and it falls into local maxima easily. To address this drawback, a parameter ε is added to acquisition function PI such that

difference between values of next sample point $\hat{x}$ and incumbent solution x^* is equal and greater than ε. Hence, the PI function is:

$$\mathrm{PI}(\hat{x}) = Pr(f(\hat{x}) \geq f(x^*) + \varepsilon) = \phi(\frac{\mu_f(\hat{x}) - f(x^*) - \varepsilon}{\Sigma_f(\hat{x})}).$$

Expected Improvement maximizes the expected degree of improvement over the incumbent solution x^*, where the degree of improvement is $I(\hat{x}) = \max\{0, f(\hat{x}) - f(x^*)\}$ as the difference between values of next sample point $\hat{x}$ and incumbent solution x^*[4]. Therefore, the expected improvement acquisition function is defnined as

$$\mathrm{EI}(\hat{x}) = E(I) = \Sigma_f(\hat{x})[Z\phi(Z) + \varphi(Z)],$$

where Z equals to $\frac{\mu_f(\hat{x}) - f(x^*)}{\Sigma_f(\hat{x})}$, and $\varphi(.)$ is probability density function of the standard normal distribution. Comparison between PI function and EI function shows that EI function is less prone to fall into the local maxima.

GP Upper Confidence Bound as the recent acquisition function is a trade-off between exploiting lower confidence zone or exploring near incumbent solution x^* with a higher expected value[4]. This acquisition function is defined as

$$\mathrm{UCB}(\hat{x}) = \mu_f(\hat{x}) - K\Sigma_f(\hat{x}),$$

where parameter K indicates the balance between exploitation and exploration.

Appendix 2

Figures 1-25 present complementary information to the autoregressive model's performance on the estimating weighted average parameters values in the proposed framework.

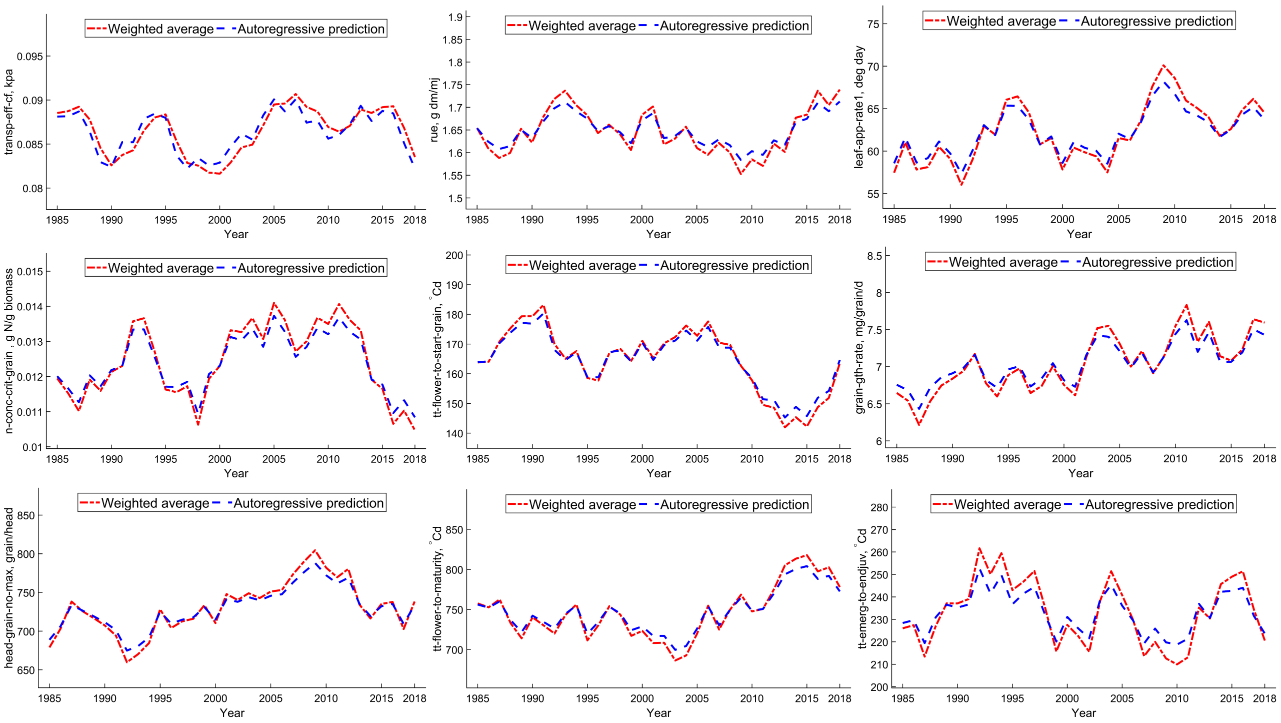

Figure 1. The autoregressive model's performance in predicting weighted average parameter values for nine parameters at location 1, Logan, Illinois.

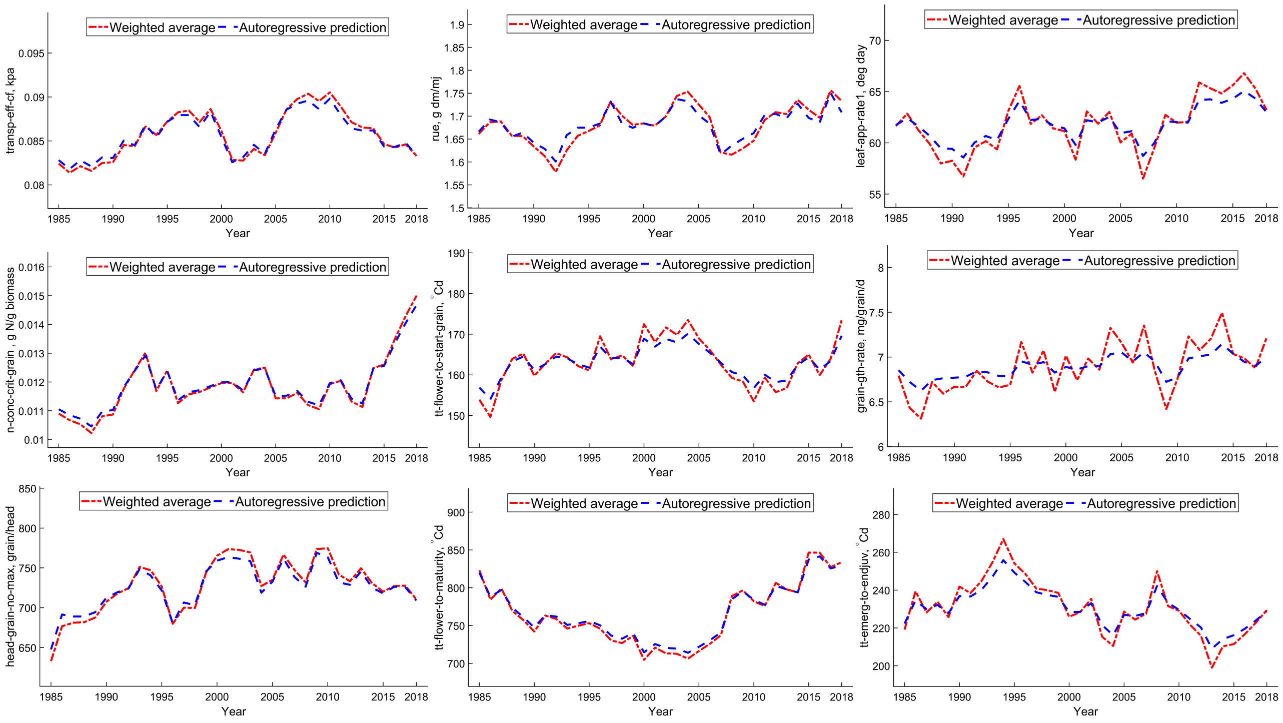

Figure 2. The autoregressive model's performance in predicting weighted average parameter values for nine parameters at location 2, Logan, Illinois.

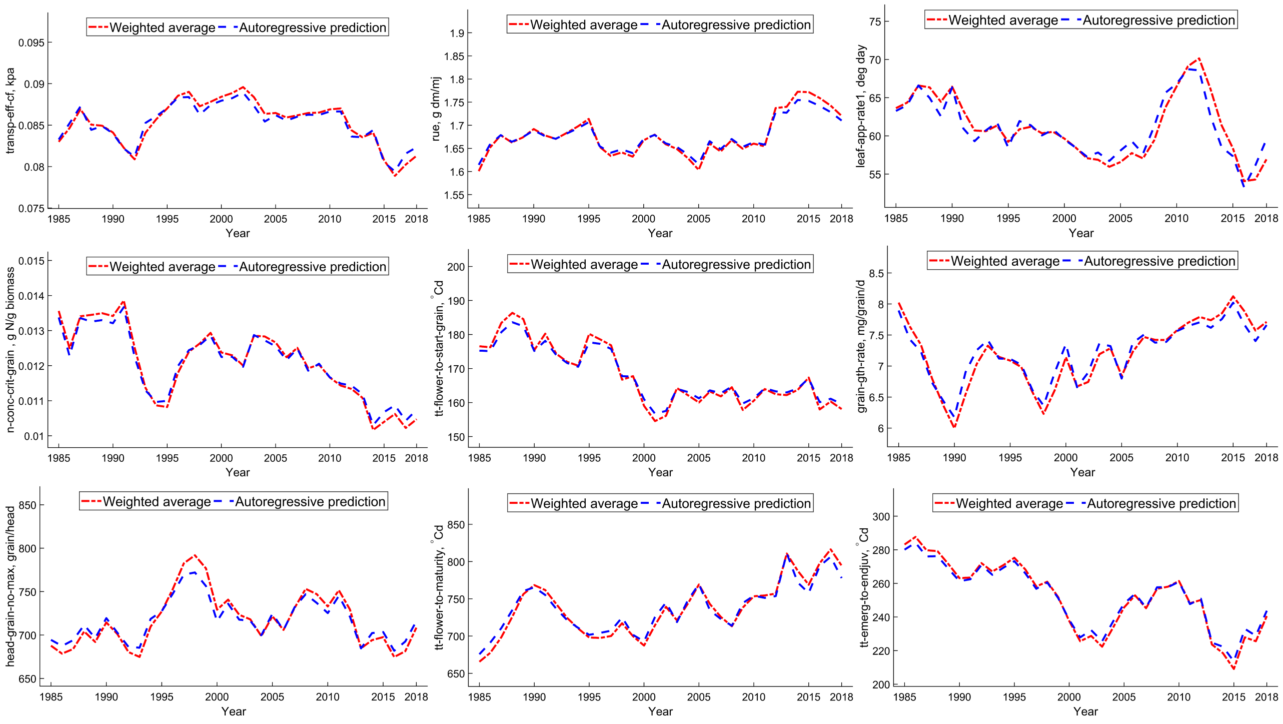

Figure 3. The autoregressive model's performance in predicting weighted average parameter values for nine parameters at location 3, Logan, Illinois.

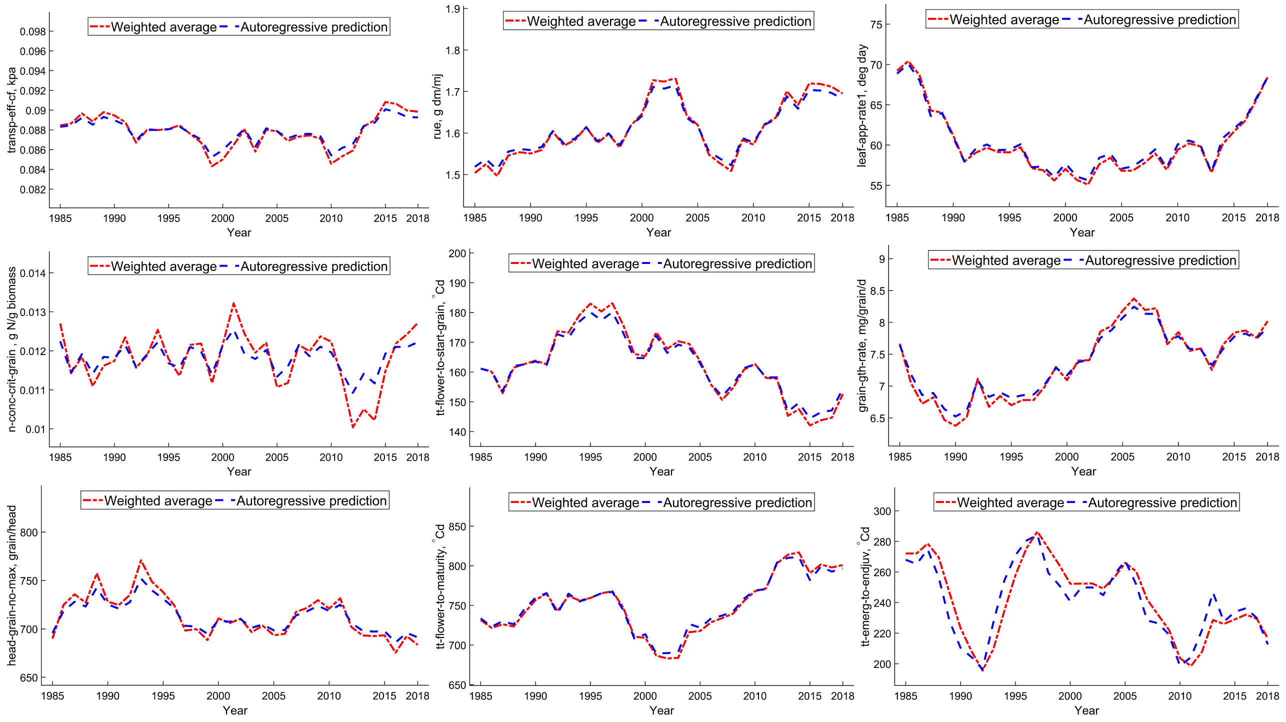

Figure 4. The autoregressive model's performance in predicting weighted average parameter values for nine parameters at location 4, Logan, Illinois.

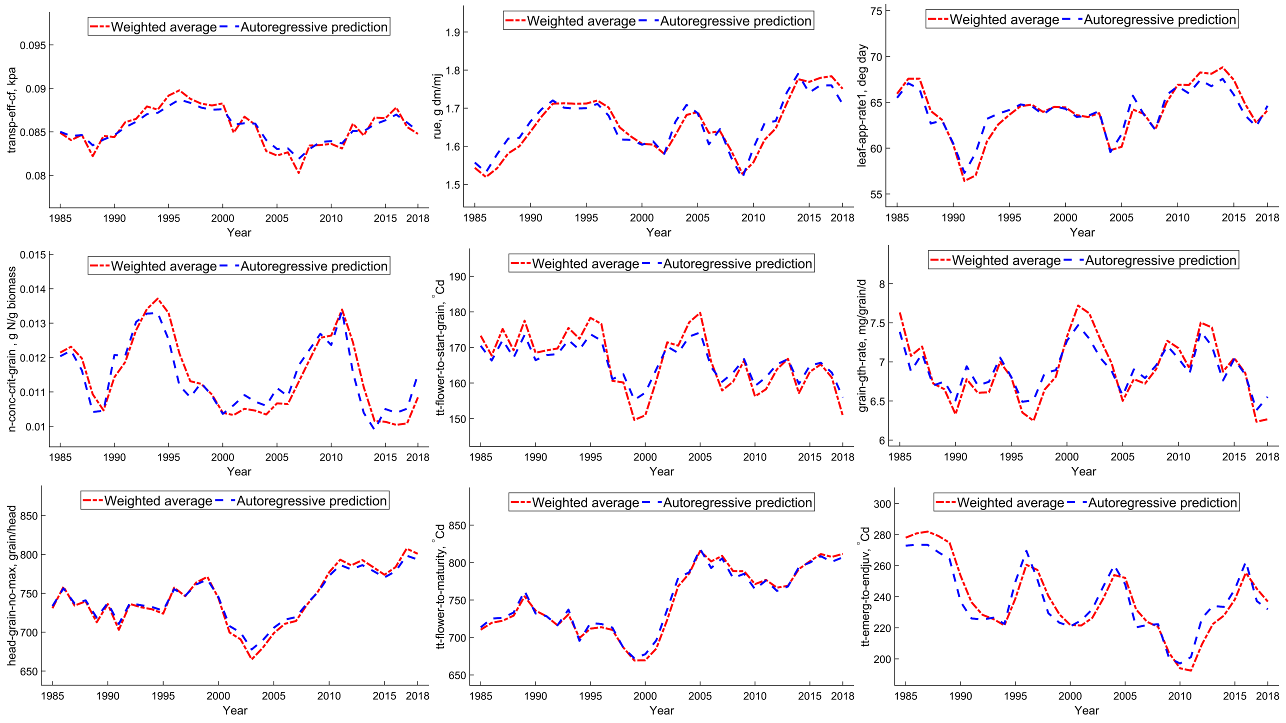

Figure 5. The autoregressive model's performance in predicting weighted average parameter values for nine parameters at location 5, Logan, Illinois.

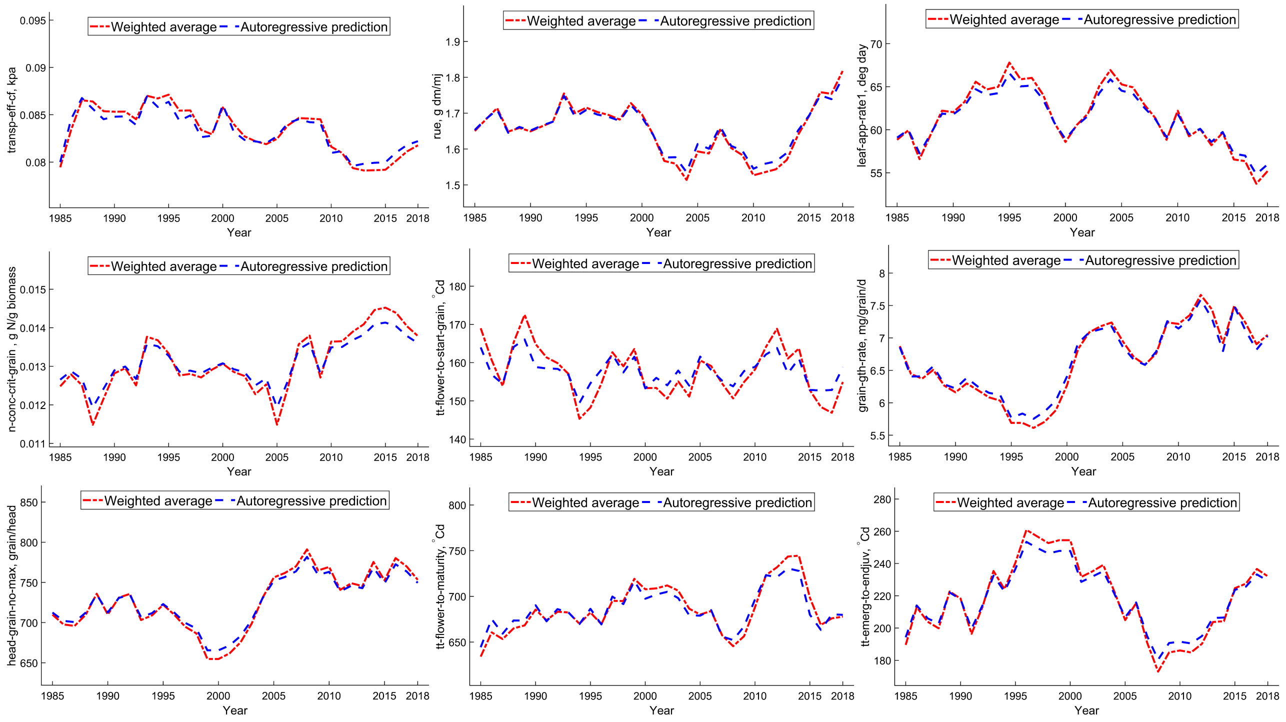

Figure 6. The autoregressive model's performance in predicting weighted average parameter values for nine parameters at location 1, Greene, Indiana.

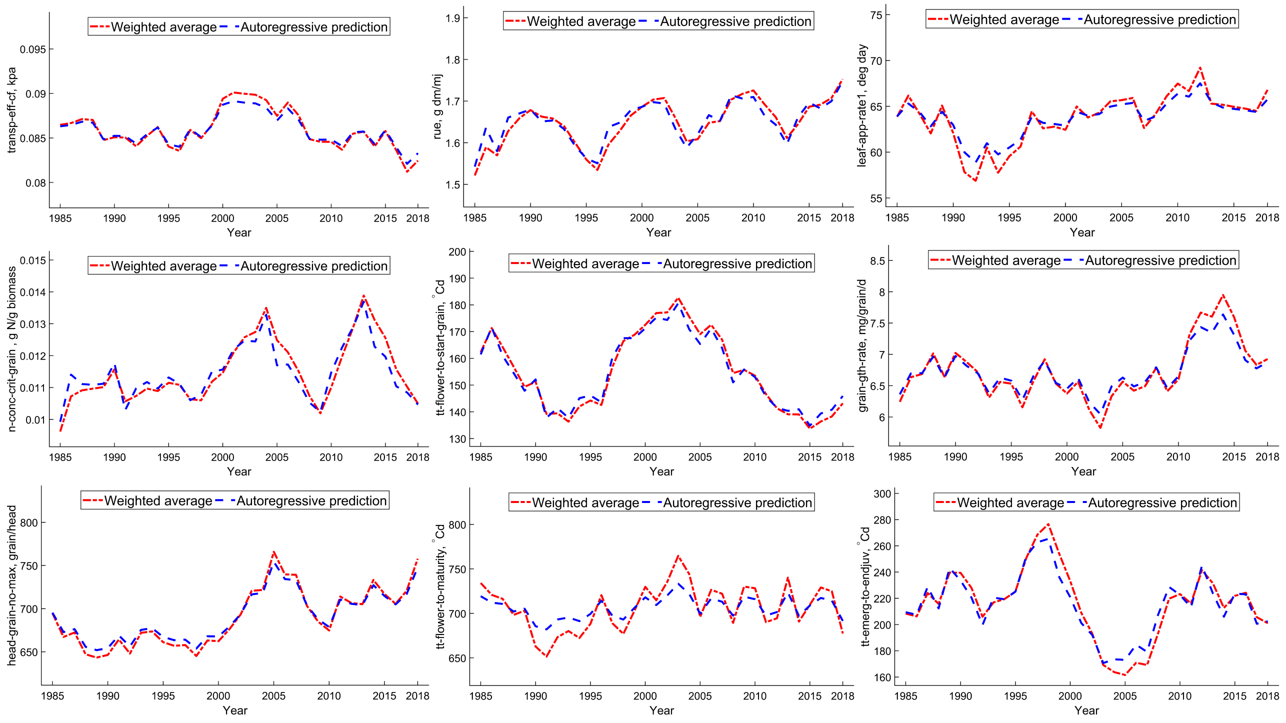

Figure 7. The autoregressive model's performance in predicting weighted average parameter values for nine parameters at location 2, Greene, Indiana.

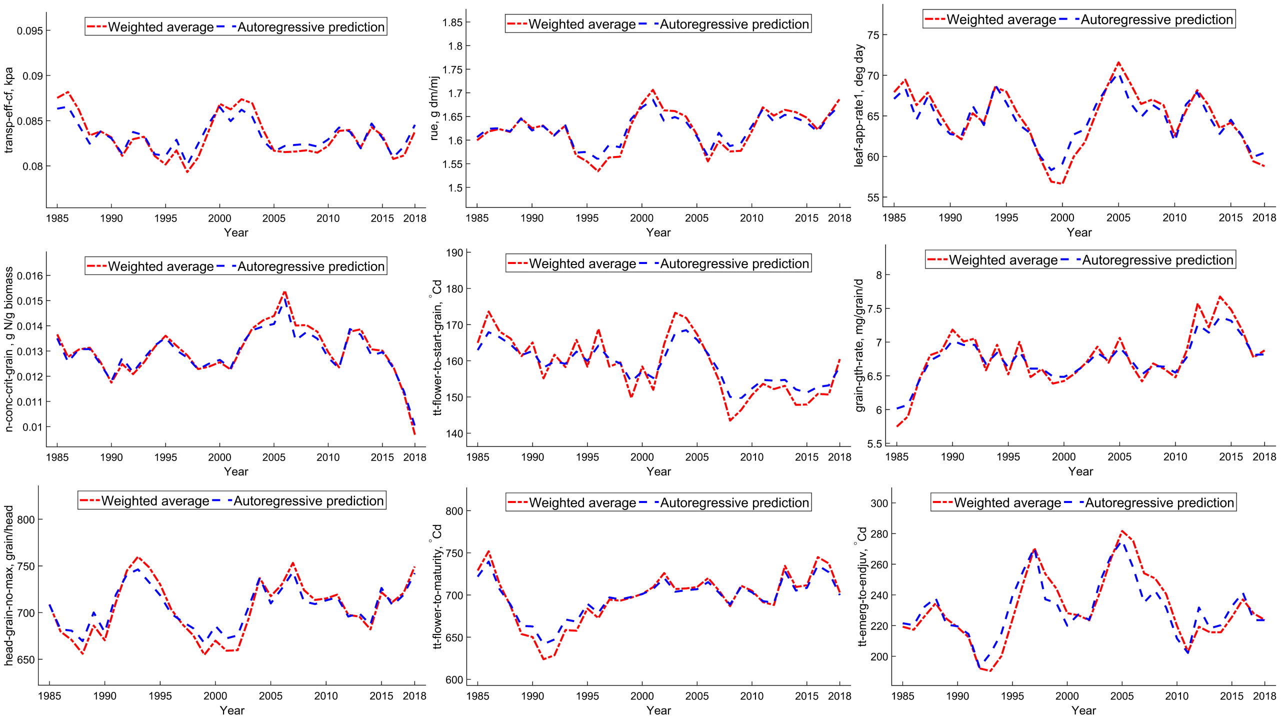

Figure 8. The autoregressive model's performance in predicting weighted average parameter values for nine parameters at location 3, Greene, Indiana.

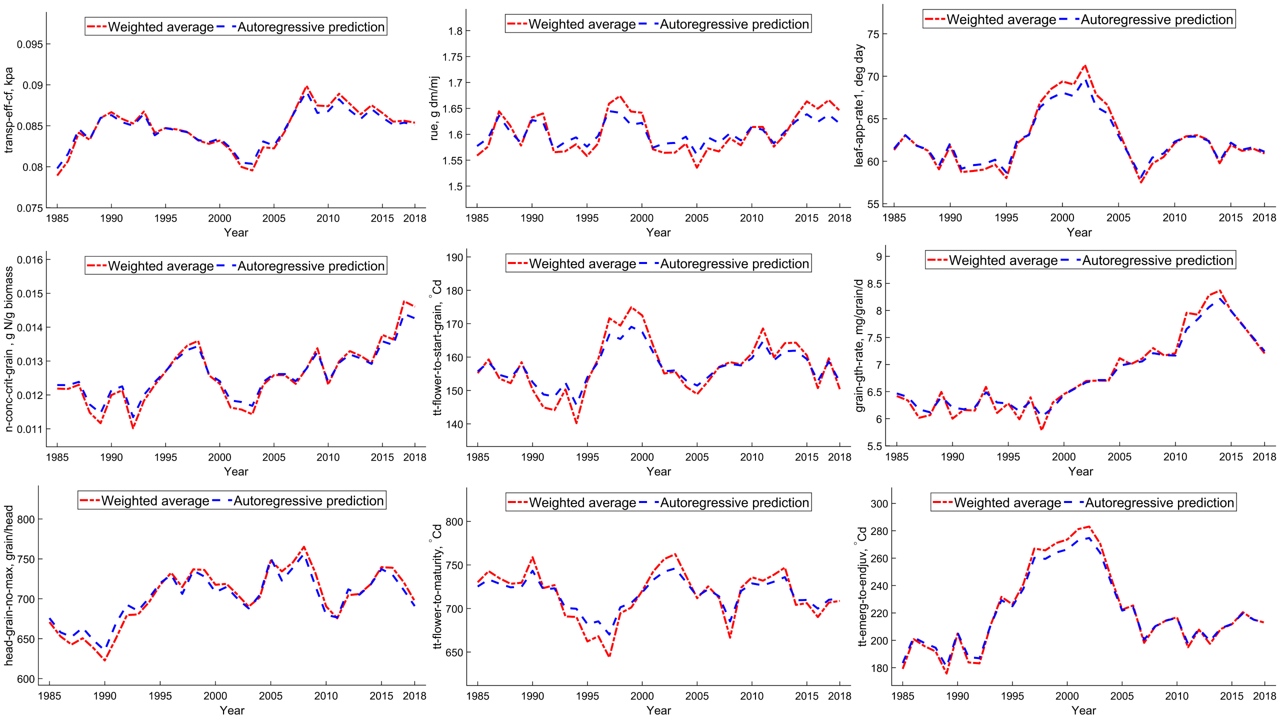

Figure 9. The autoregressive model's performance in predicting weighted average parameter values for nine parameters at location 4, Greene, Indiana.

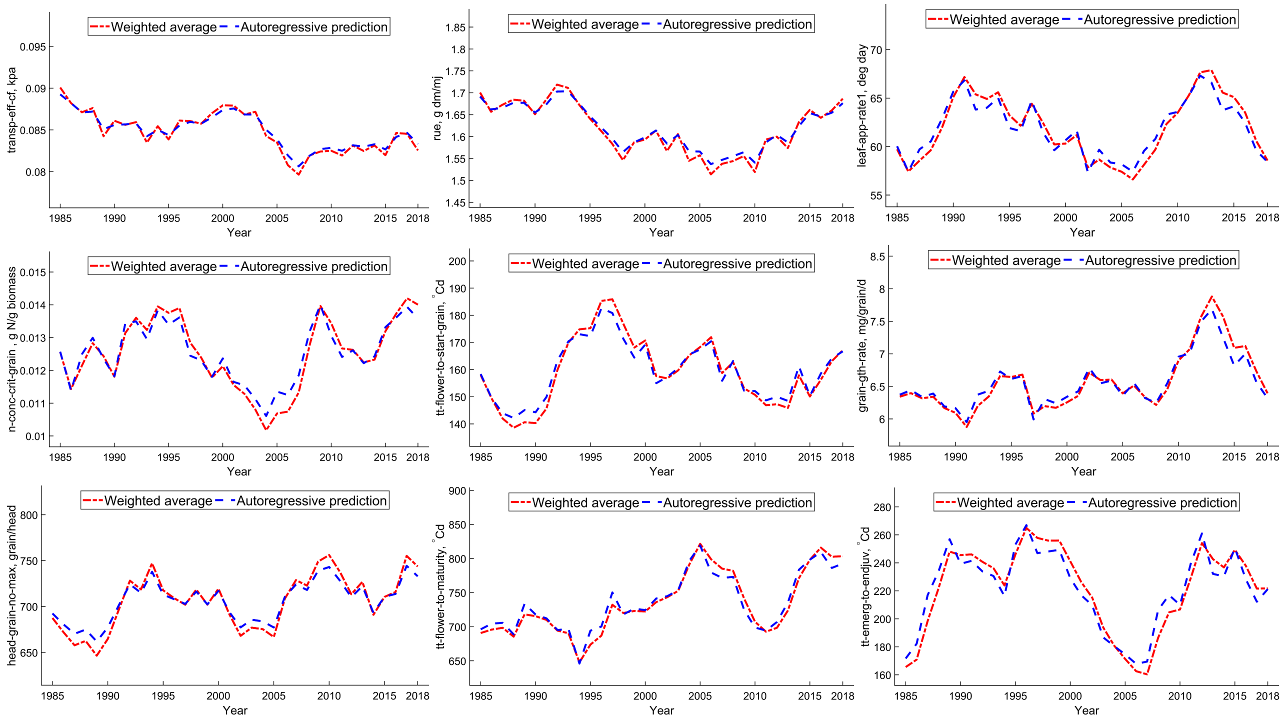

Figure 10. The autoregressive model's performance in predicting weighted average parameter values for nine parameters at location 5, Greene, Indiana.

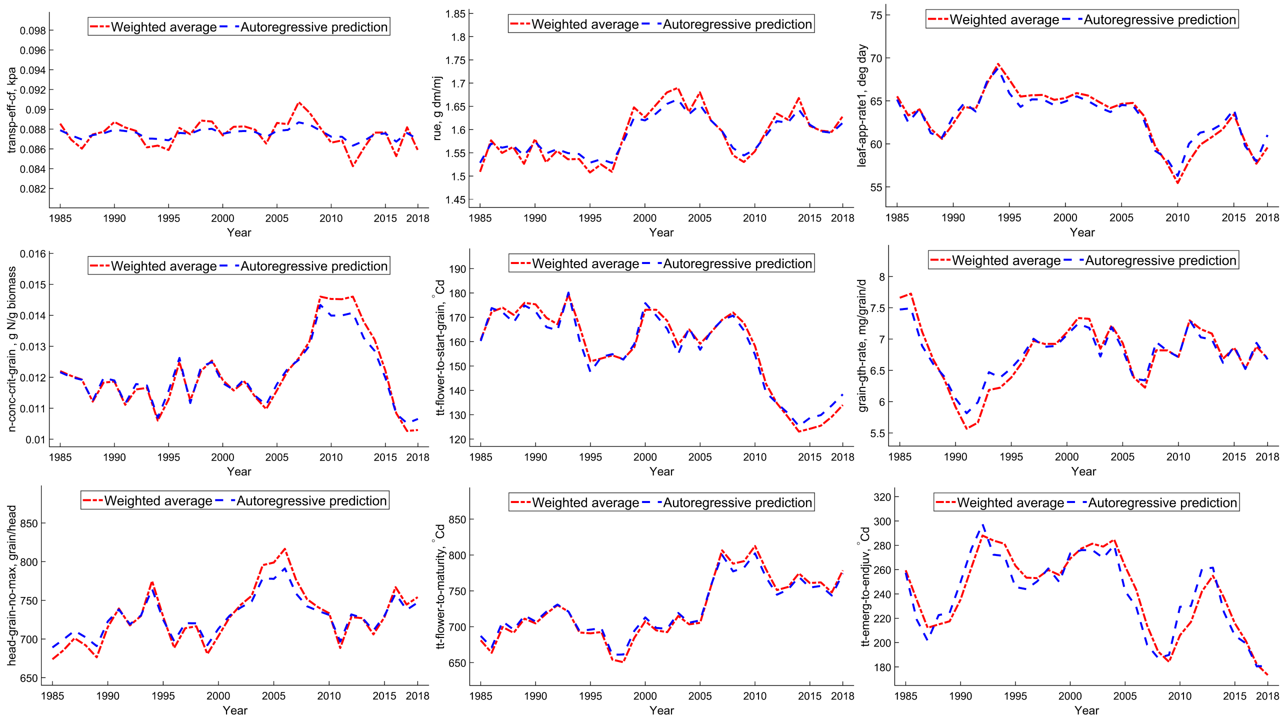

Figure 11. The autoregressive model's performance in predicting weighted average parameter values for nine parameters at location 1, Keokuk, Iowa.

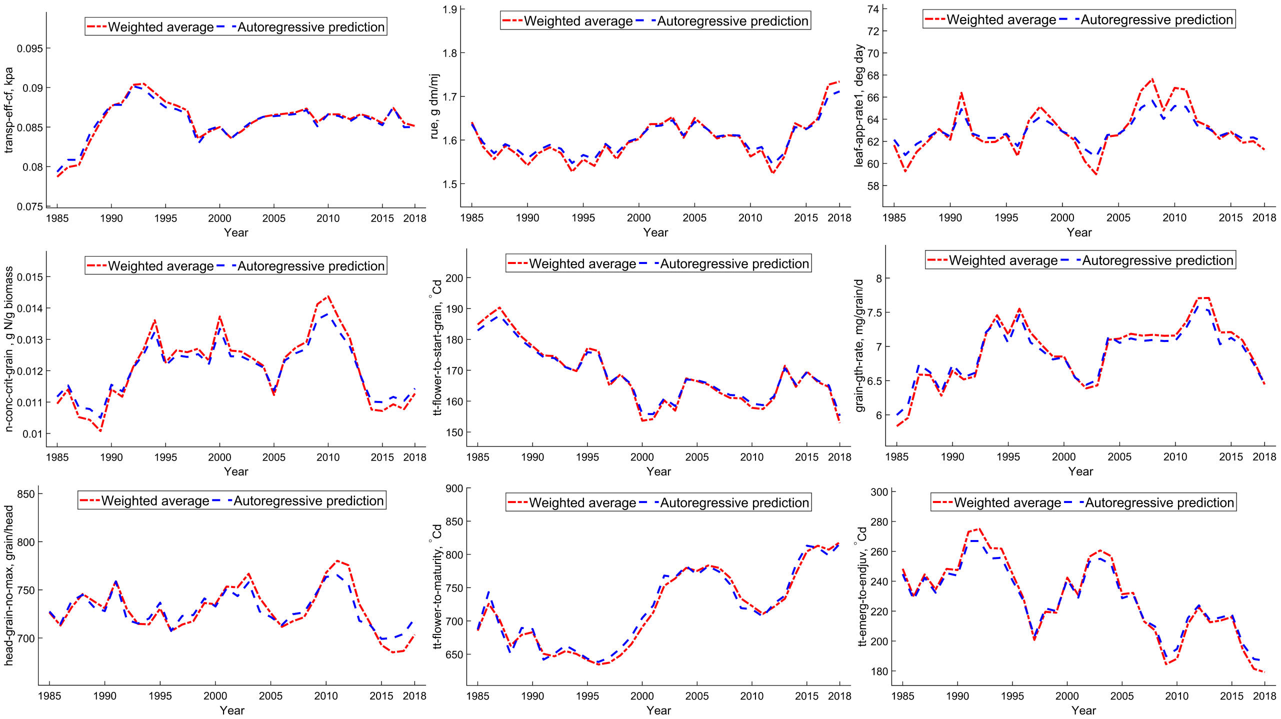

Figure 12. The autoregressive model's performance in predicting weighted average parameter values for nine parameters at location 2, Keokuk, Iowa.

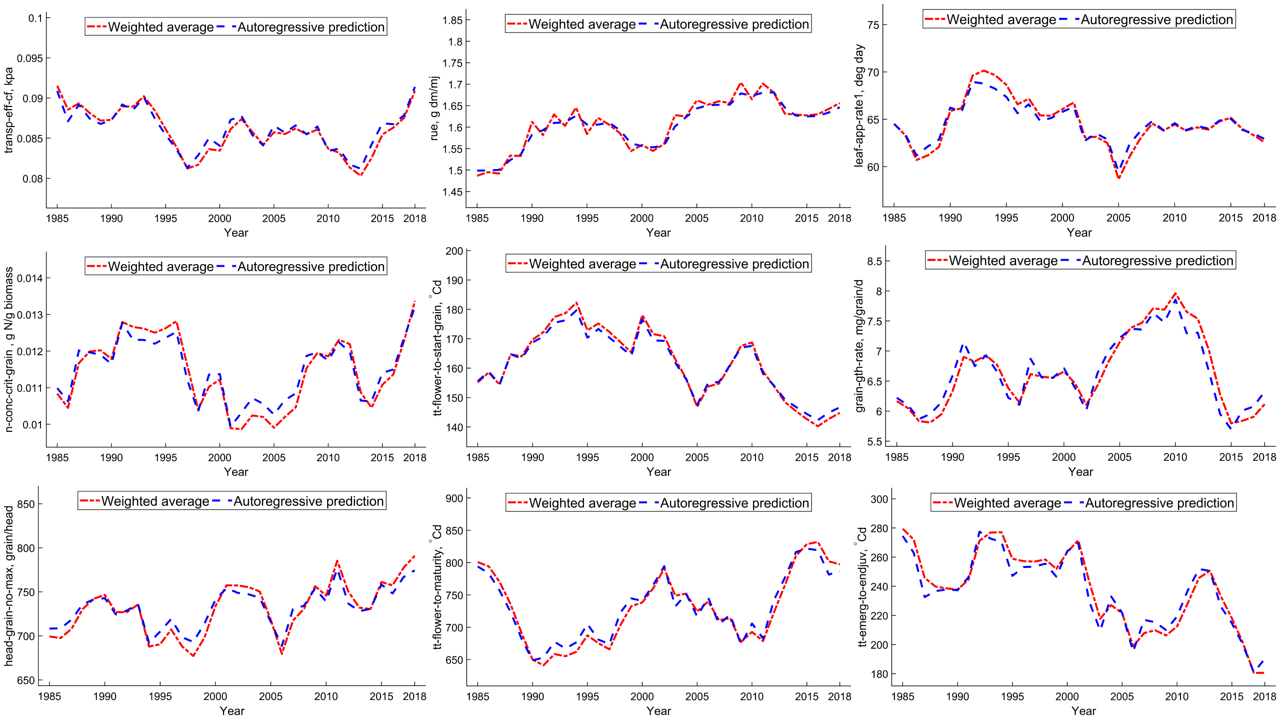

Figure 13. The autoregressive model's performance in predicting weighted average parameter values for nine parameters at location 3, Keokuk, Iowa.

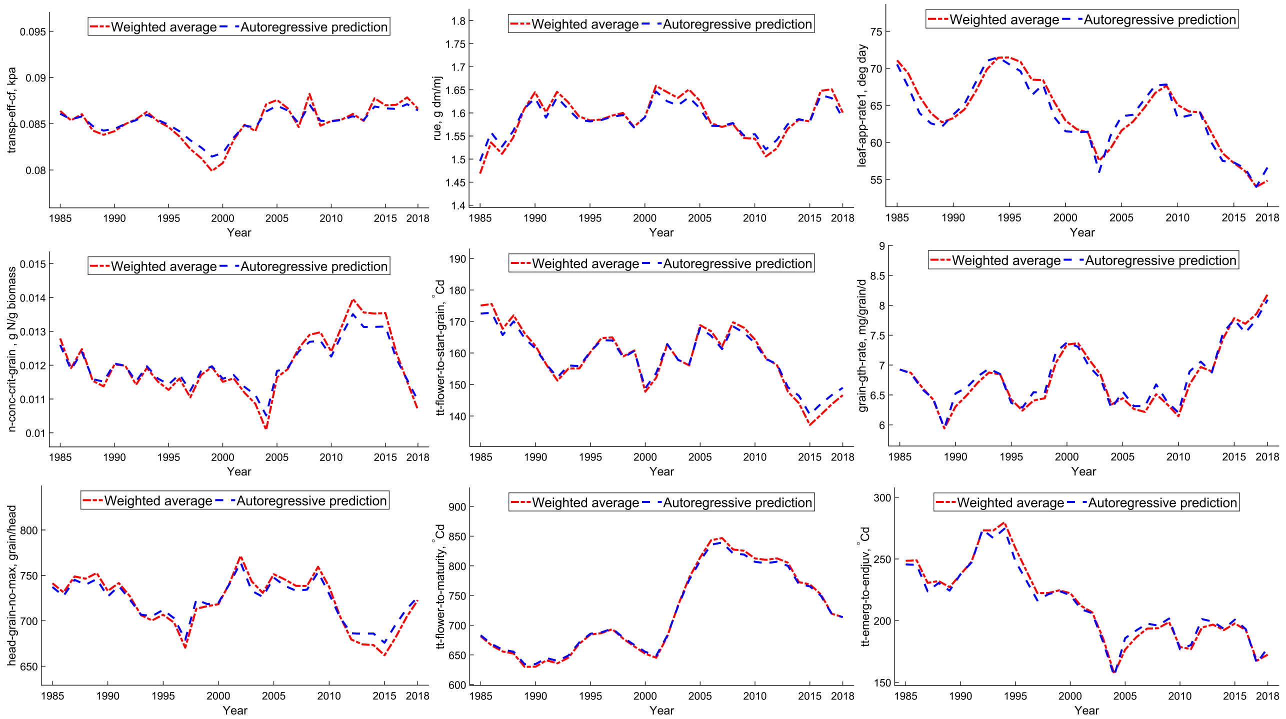

Figure 14. The autoregressive model's performance in predicting weighted average parameter values for nine parameters at location 4, Keokuk, Iowa.

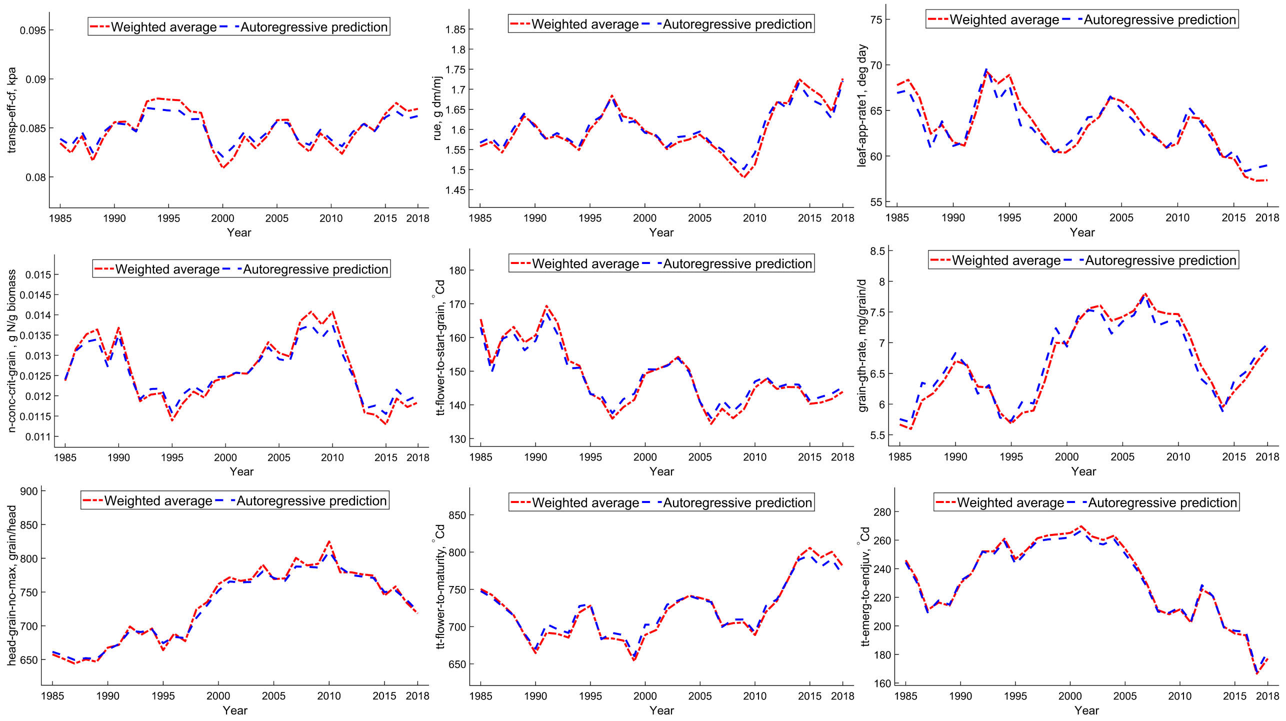

Figure 15. The autoregressive model's performance in predicting weighted average parameter values for nine parameters at location 5, Keokuk, Iowa.

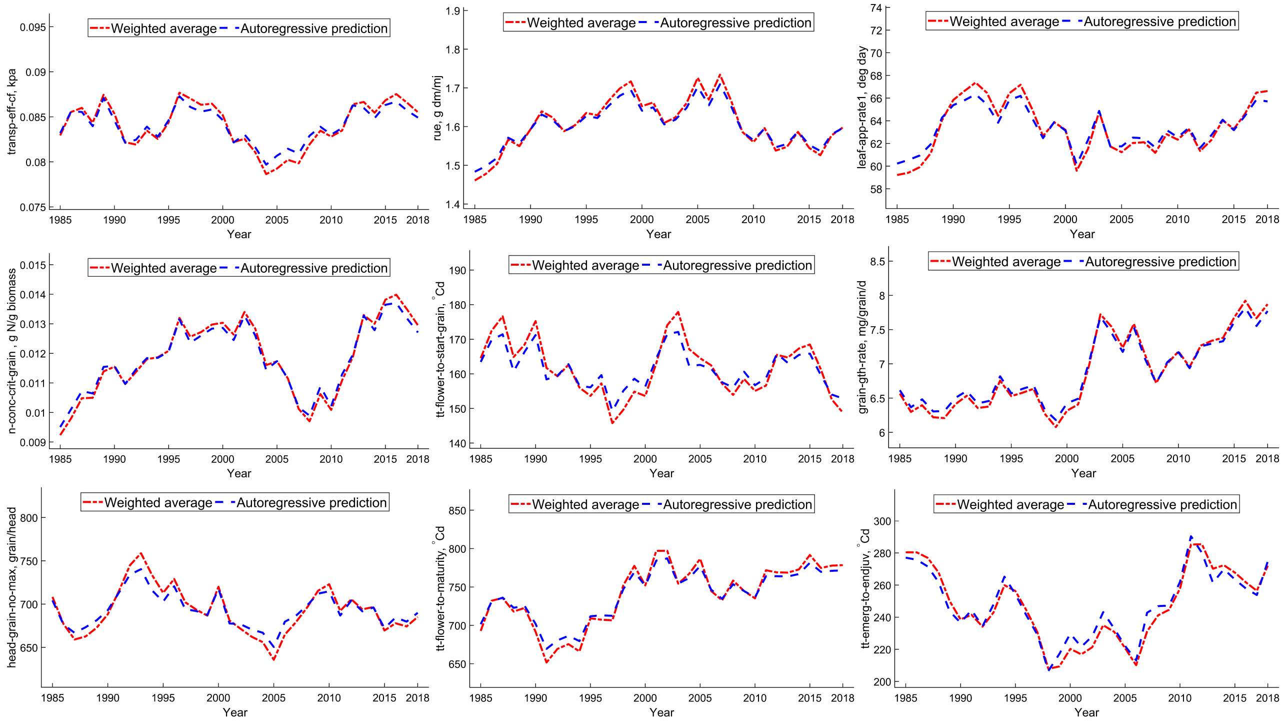

Figure 16. The autoregressive model's performance in predicting weighted average parameter values for nine parameters at location 1, Boone, Iowa.

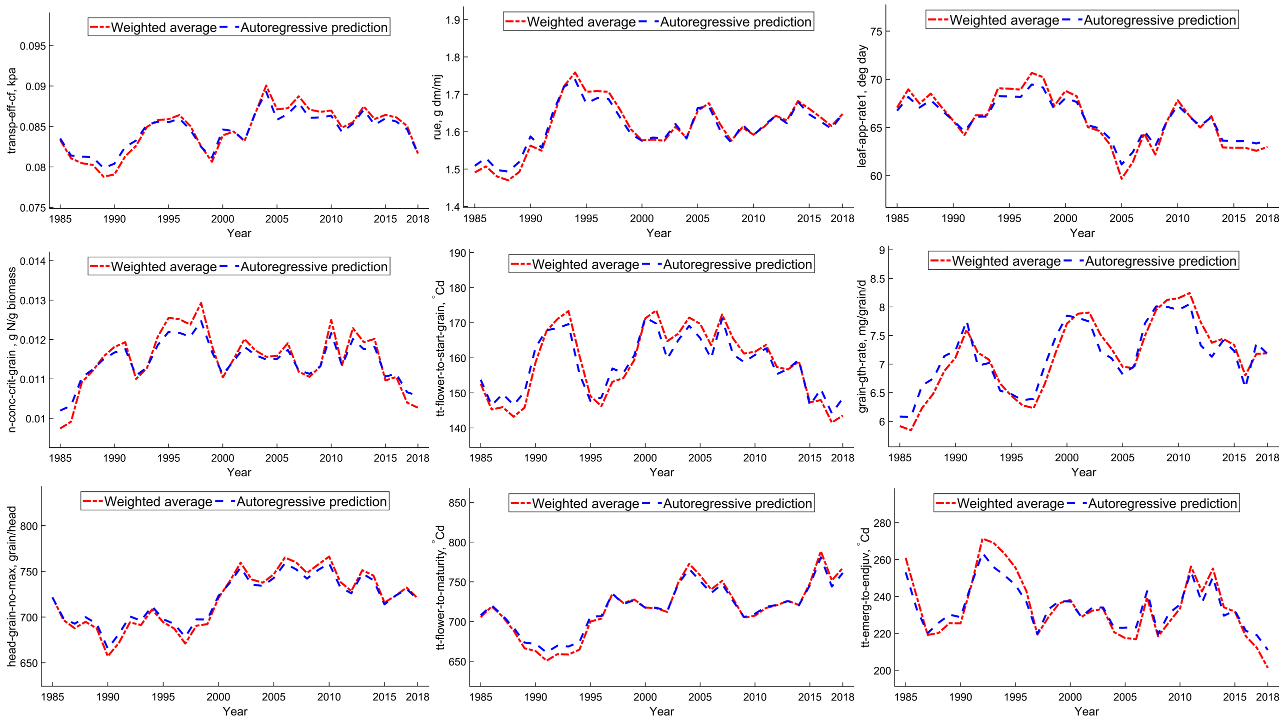

Figure 17. The autoregressive model's performance in predicting weighted average parameter values for nine parameters at location 2, Boone, Iowa.

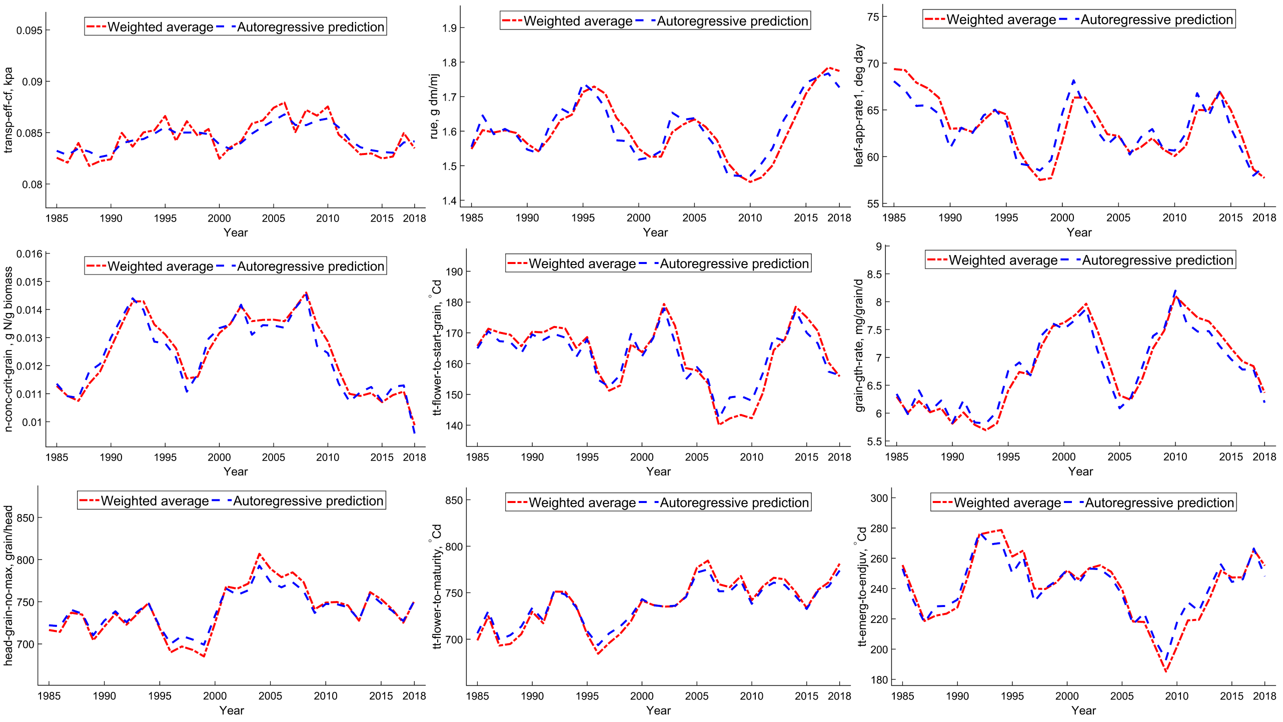

Figure 18. The autoregressive model's performance in predicting weighted average parameter values for nine parameters at location 3, Boone, Iowa.

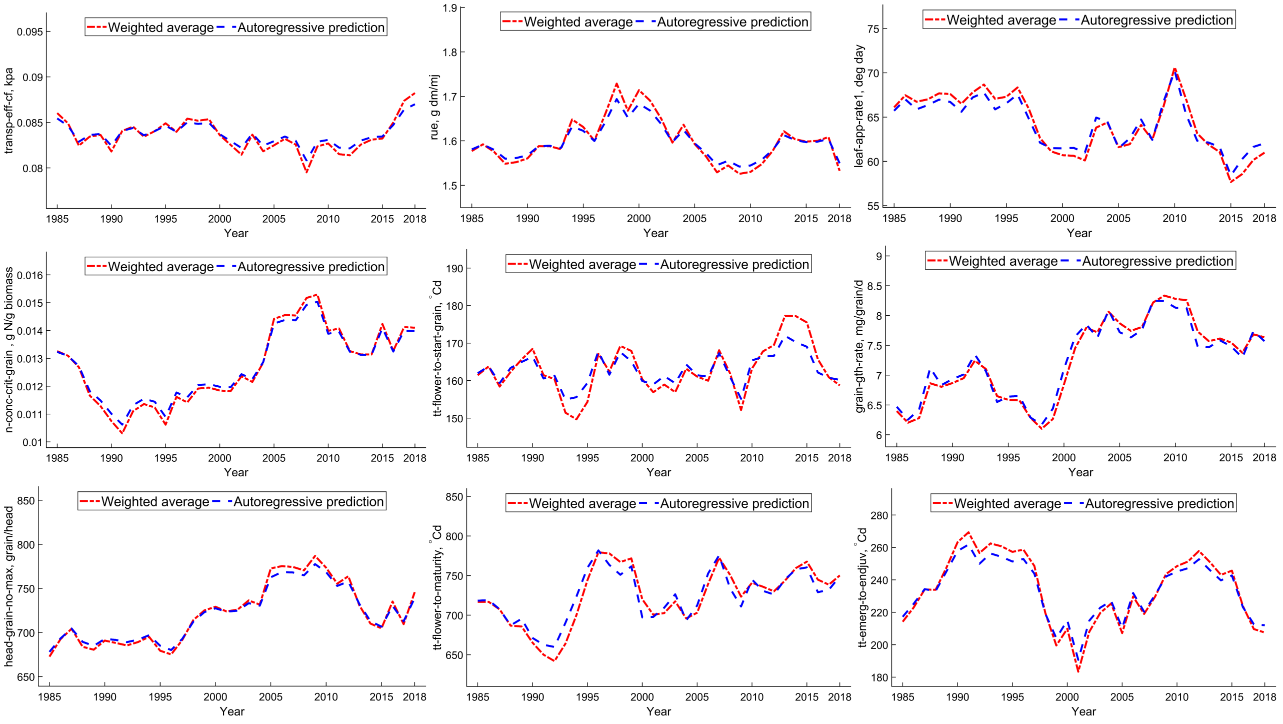

Figure 19. The autoregressive model's performance in predicting weighted average parameter values for nine parameters at location 4, Boone, Iowa.

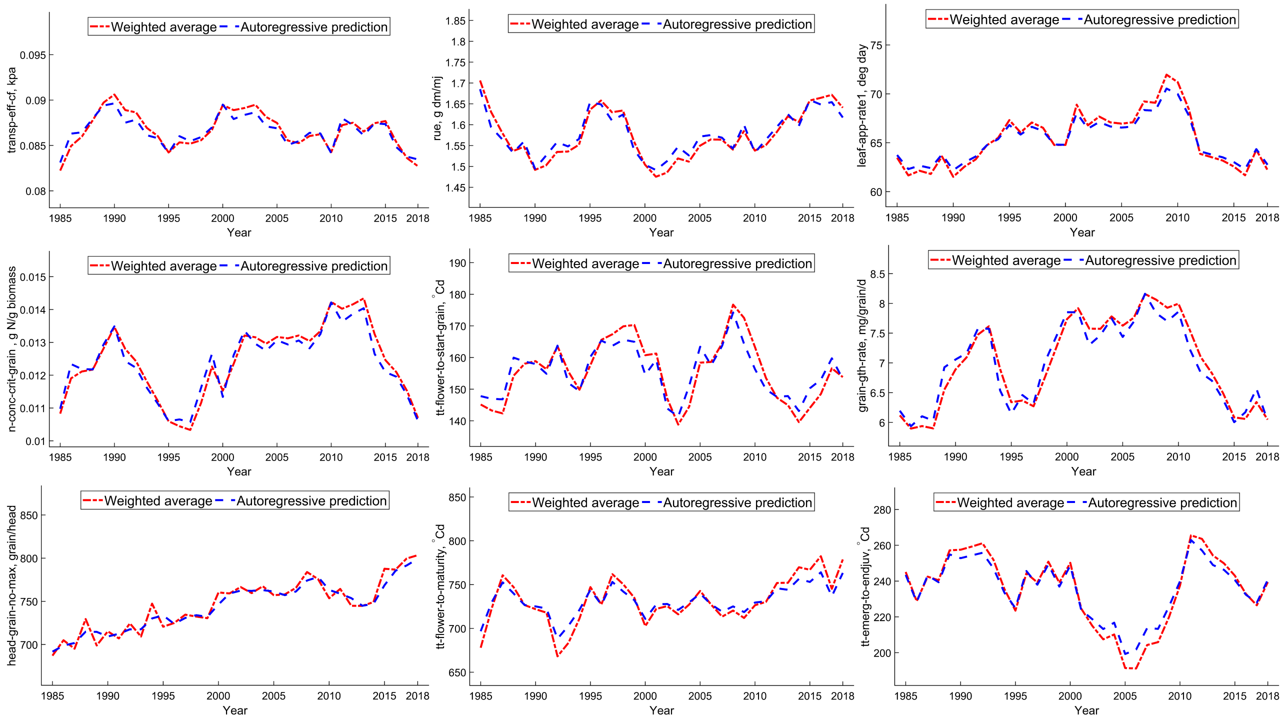

Figure 20. The autoregressive model's performance in predicting weighted average parameter values for nine parameters at location 5, Boone, Iowa.

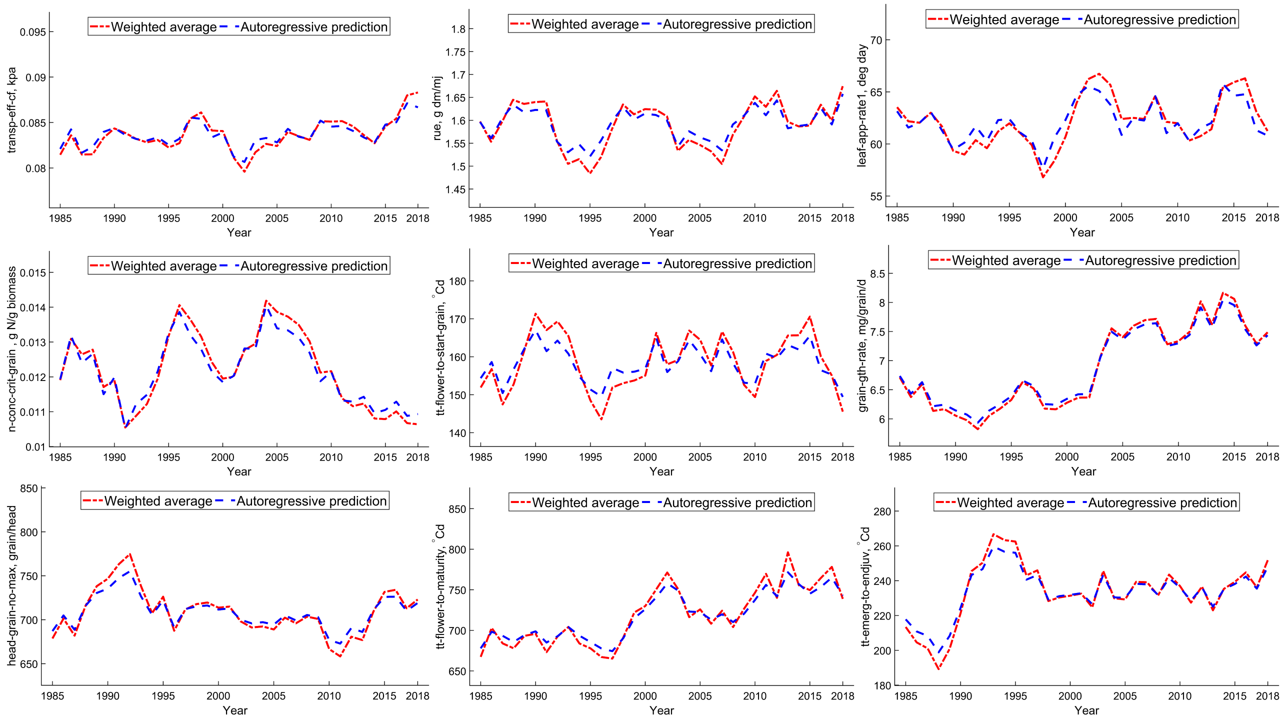

Figure 21. The autoregressive model's performance in predicting weighted average parameter values for nine parameters at location 1, Obrien, Iowa.

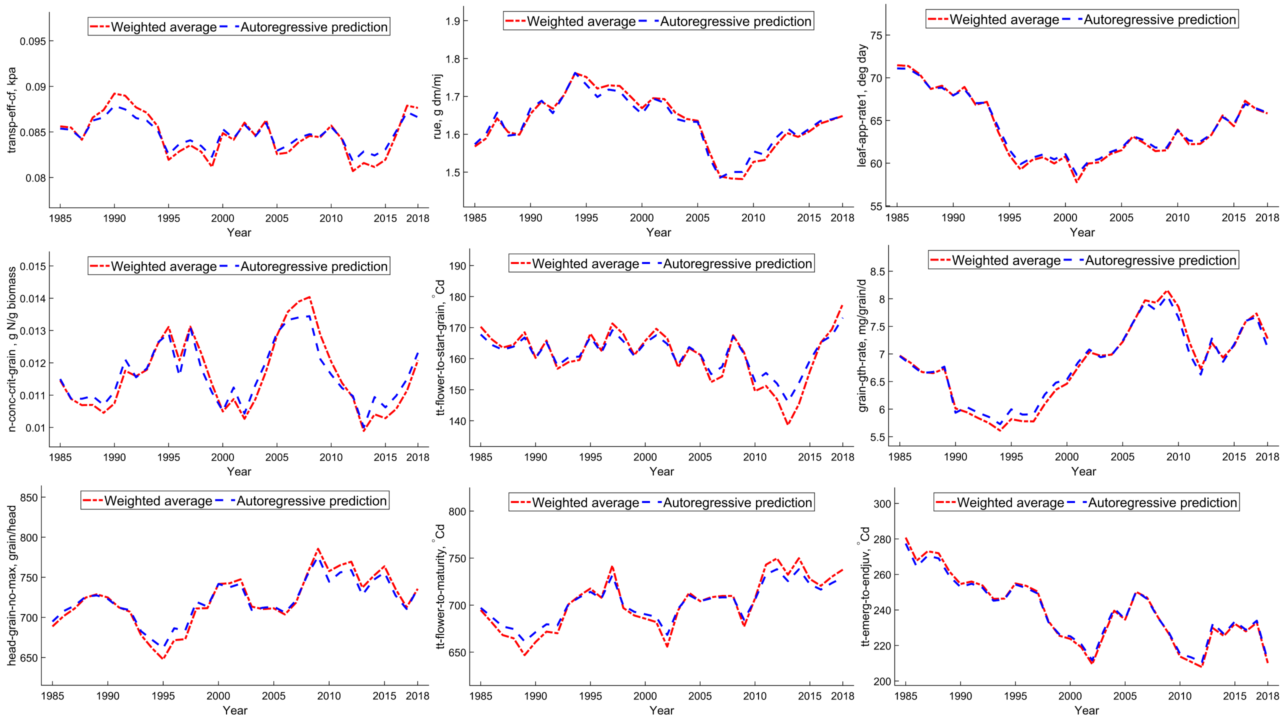

Figure 22. The autoregressive model's performance in predicting weighted average parameter values for nine parameters at location 2, Obrien, Iowa.

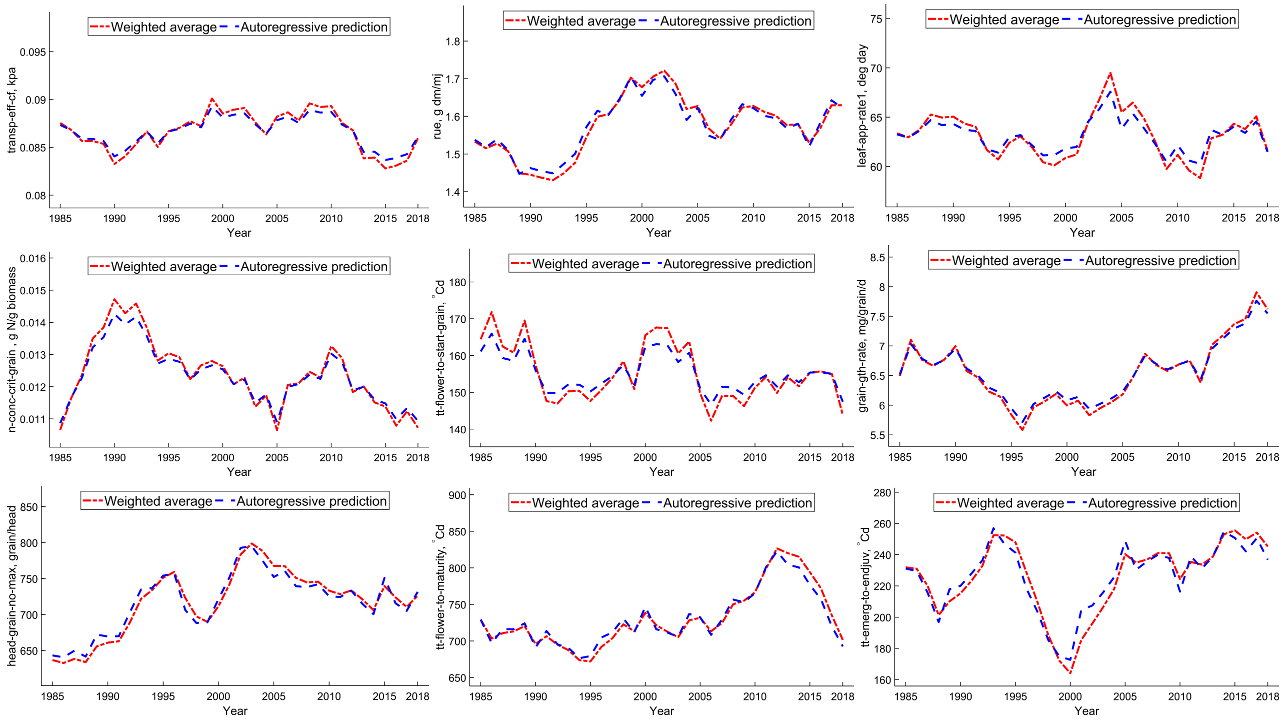

Figure 23. The autoregressive model's performance in predicting weighted average parameter values for nine parameters at location 3, Obrien, Iowa.

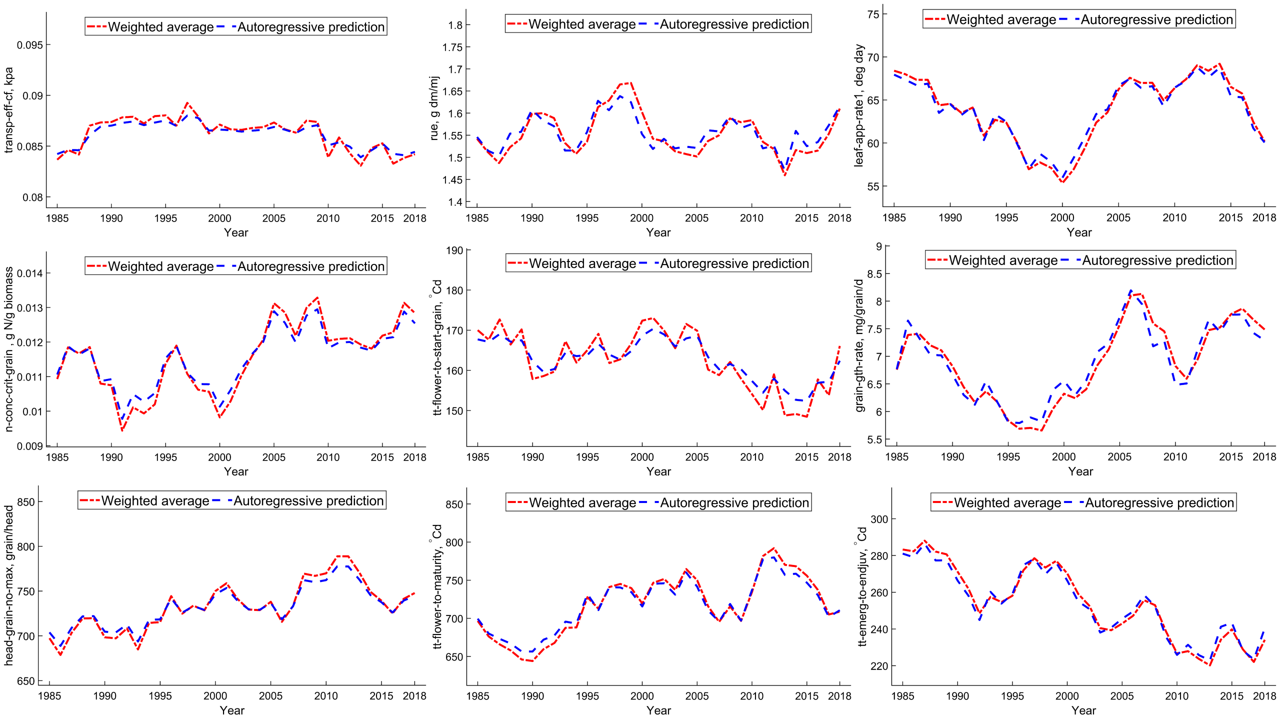

Figure 24. The autoregressive model's performance in predicting weighted average parameter values for nine parameters at location 4, Obrien, Iowa.

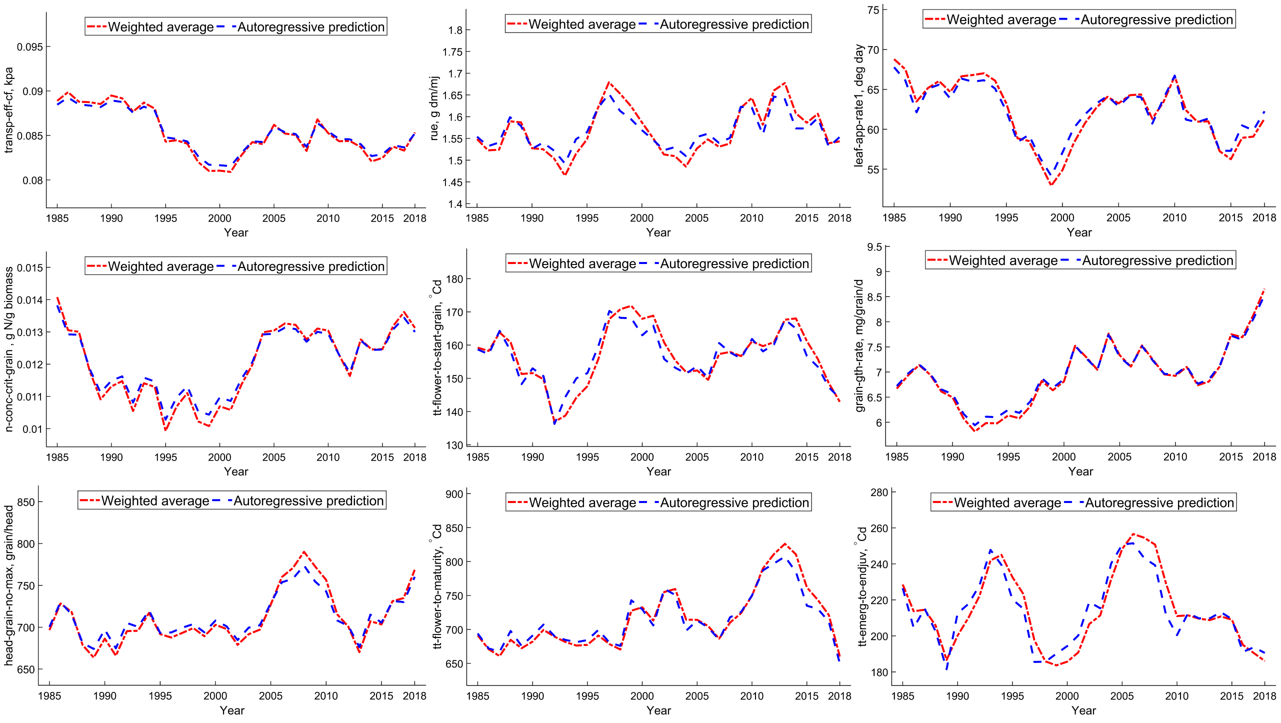

Figure 25. The autoregressive model's performance in predicting weighted average parameter values for nine parameters at location 5, Obrien, Iowa.

References

1. Brochu, E., Cora, V. M. & De Freitas, N. A tutorial on Bayesian optimization of expensive cost functions, with application to active user modeling and hierarchical reinforcement learning. *ArXiv preprint arXiv:1012.2599* (2010).
2. Gupta, A. K., Smith, K. G. & Shalley, C. E. The interplay between exploration and exploitation. *Acad. Manag. J.* **49**, 693–706 (2006).
3. Williams, C. K. & Rasmussen, C. E. *Gaussian processes for machine learning*, vol. 2 (MIT press Cambridge, MA, 2006).
4. Wu, J. *et al.* Hyperparameter optimization for machine learning models based on Bayesian optimization. *J. Electron. Sci. Technol.* **17**, 26–40 (2019).
